# Soluble immune checkpoint factors reflect exhaustion of antitumor immunity and response to PD-1 blockade

**DOI:** 10.1172/JCI168318

**Published:** 2024-04-01

**Authors:** Hidetoshi Hayashi, Kenji Chamoto, Ryusuke Hatae, Takashi Kurosaki, Yosuke Togashi, Kazuya Fukuoka, Megumi Goto, Yasutaka Chiba, Shuta Tomida, Takayo Ota, Koji Haratani, Takayuki Takahama, Junko Tanizaki, Takeshi Yoshida, Tsutomu Iwasa, Kaoru Tanaka, Masayuki Takeda, Tomoko Hirano, Hironori Yoshida, Hiroaki Ozasa, Yuichi Sakamori, Kazuko Sakai, Keiko Higuchi, Hitoshi Uga, Chihiro Suminaka, Toyohiro Hirai, Kazuto Nishio, Kazuhiko Nakagawa, Tasuku Honjo

**Affiliations:** 1 Department of Medical Oncology, Kindai University Faculty of Medicine, Osaka-Sayama, Japan.; 2 Department of Immunology and Genomic Medicine, Center for Cancer Immunotherapy and Immunobiology, Graduate School of Medicine, Kyoto University, Kyoto, Japan.; 3 Department of Immuno-Oncology PDT, Kyoto University Graduate School of Medicine, Kyoto, Japan.; 4 Department of Genome Biology, Kindai University Faculty of Medicine, Osaka-Sayama, Japan.; 5 Department of Tumor Microenvironment, Faculty of Medicine, Dentistry, and Pharmaceutical Sciences, Okayama University, Okayama, Japan.; 6 Clinical Research Center, Kindai University Hospital, Osaka-Sayama, Japan.; 7 Sysmex Corporation, Kobe, Japan.; 8 Department of Center for Comprehensive Genomic Medicine, Okayama University Hospital, Okayama, Japan.; 9 Department of Medical Oncology, Izumi City General Hospital, Izumi, Japan.; 10 Department of Cancer Genomics and Medical Oncology, Nara Medical University School of Medicine, Nara, Japan.; 11 Department of Respiratory Medicine, Graduate School of Medicine, Kyoto University, Kyoto, Japan.; 12 Department of Clinical Oncology, Kyoto University Hospital, Kyoto, Japan.

**Keywords:** Oncology, Immunotherapy, Lung cancer, T cells

## Abstract

**BACKGROUND:**

Precise stratification of patients with non–small cell lung cancer (NSCLC) is needed for appropriate application of PD-1/PD-L1 blockade therapy.

**METHODS:**

We measured soluble forms of the immune-checkpoint molecules PD-L1, PD-1, and CTLA-4 in plasma of patients with advanced NSCLC before PD-1/PD-L1 blockade. A prospective biomarker-finding trial (cohort A) included 50 previously treated patients who received nivolumab. A retrospective observational study was performed for patients treated with any PD-1/PD-L1 blockade therapy (cohorts B and C), cytotoxic chemotherapy (cohort D), or targeted therapy (cohort E). Plasma samples from all patients were assayed for soluble immune-checkpoint molecules with a highly sensitive chemiluminescence-based assay.

**RESULTS:**

Nonresponsiveness to PD-1/PD-L1 blockade therapy was associated with higher concentrations of these soluble immune factors among patients with immune-reactive (hot) tumors. Such an association was not apparent for patients treated with cytotoxic chemotherapy or targeted therapy. Integrative analysis of tumor size, PD-L1 expression in tumor tissue (tPD-L1), and gene expression in tumor tissue and peripheral CD8^+^ T cells revealed that high concentrations of the 3 soluble immune factors were associated with hyper or terminal exhaustion of antitumor immunity. The combination of soluble PD-L1 (sPD-L1) and sCTLA-4 efficiently discriminated responsiveness to PD-1/PD-L1 blockade among patients with immune-reactive tumors.

**CONCLUSION:**

Combinations of soluble immune factors might be able to identify patients unlikely to respond to PD-1/PD-L1 blockade as a result of terminal exhaustion of antitumor immunity. Our data suggest that such a combination better predicts, along with tPD-L1, for the response of patients with NSCLC.

**TRIAL REGISTRATION:**

UMIN000019674.

**FUNDING:**

This study was funded by Ono Pharmaceutical Co. Ltd. and Sysmex Corporation.

## Abstract 2

Combinations of soluble immune factors identifies patients that are unlikely to respond to PD-1/PD-L1 blockade because of terminal exhaustion of antitumor immunity.

## Introduction

Targeting of the immune system has provided clinical benefit for individuals with advanced solid tumors, including non–small cell lung cancer (NSCLC) (1). Currently approved immune-checkpoint inhibitors (ICIs) include monoclonal antibodies that target cytotoxic T lymphocyte–associated protein–4 (CTLA-4) or programmed cell death–1 (PD-1) pathways (2–5). Expression of the PD-1 ligand PD-L1 in tumor tissue (tPD-L1) is the most well-established biomarker for treatment with antibodies to PD-1 or to PD-L1 in patients with advanced NSCLC. However, the identification of additional biomarkers is necessary because of the insufficient prediction value of tPD-L1, which is attributed, in part, to heterogeneity of tPD-L1 expression (6).

Soluble PD-1 (sPD-1), sCTLA-4, and sPD-L1 have been explored as potential biomarkers for ICI therapy. sPD-1 is released into circulation mainly in the form of a splice variant that lacks the transmembrane domain encoded by exon 3 of the PD-1 gene, although the possibility of proteolytic cleavage of PD-1 in the cell membrane cannot be excluded (7, 8). sPD-1 activates antitumor immunity by interrupting the interaction of PD-1 with PD-L1 in animal models (8, 9). However, a high serum concentration of sPD-1 before treatment has been associated with a low efficacy of PD-1 blockade therapy in patients with melanoma or NSCLC (10, 11).

sPD-L1 is generated predominantly by proteolytic cleavage of PD-L1 in the cell membrane, although splice variant–derived sPD-L1 has also been identified (12–14). Given that PD-L1 is aberrantly expressed on various cell types, including macrophage-lineage cells and tumor cells in cancer patients, the source of sPD-L1 in such individuals likely reflects this expression pattern (15). Whereas the function of sPD-L1 in immunity remains unclear (14), a high concentration of sPD-L1 in blood has consistently been found to indicate unresponsiveness to PD-1 or PD-L1–blockade therapy in clinical studies (10, 16–18). The combination of sPD-1 and sPD-Ll was shown to be more predictive for PD-1 blockade therapy than was either marker alone (10, 11).

sCTLA-4 is thought to be derived from a splice variant that lacks the transmembrane domain encoded by exon 3 of the CTLA-4 gene (19, 20). It is potentially produced by T cells, B cells, and macrophages, and has been shown to inhibit immune reactions (19–21). Although few studies have investigated whether sCTLA-4 is able to serve as a biomarker for PD-1/PD-L1 blockade therapy, a high circulating concentration of sCTLA-4 has been associated with a positive response to therapy with the anti–CTLA-4 antibody ipilimumab (22, 23).

PD-1 and CTLA-4 are hallmarks of T cell exhaustion or overactivation. The extent of T cell exhaustion has been examined as a potential biomarker for PD-1 blockade therapy (24–27). Recent evidence has indicated that, rather than fully exhausted CD8^+^ T cells, preexhausted (progenitor exhausted) CD8^+^ T cells with proliferative capacity, such as CD8^+^ T cells positive for PD-1 and T cell factor 1 (TCF1), are the major effector cells attacking cancer cells during PD-1 blockade therapy (25, 28–33). Cancer patients with a high frequency of terminally exhausted T cells and a low frequency of progenitor exhausted T cells at tumor sites would therefore be expected to be nonresponsive to such treatment (26, 27, 29, 34, 35). However, systemic measurement of the extent of exhaustion in T cells has remained a challenge.

Among sPD-1, sPD-L1, and sCTLA-4, most previous investigations have focused on the link between sPD-L1 and clinical outcome. This focus on sPD-L1 has likely been due to the difficulty of precise measurement of low concentrations of sPD-1 and sCTLA-4 in pretreatment blood samples by commercially available ELISA kits. To address this difficulty, we developed a fully automated assay for sPD-1, sPD-L1, and sCTLA-4 that is based on chemiluminescent magnetic technology (HISCL system) and that is highly sensitive, reproducible, and precise (36).

We have now applied this HISCL system to measure sPD-1, sPD-L1, and sCTLA-4 in pretreatment plasma of patients with advanced NSCLC treated with PD-1/PD-L1 blockade therapy. Integrative analysis revealed that the combination of high sPD-L1 and high sCTLA-4 levels was indicative of poor progression-free survival (PFS) in patients with immune-reactive (hot) tumors. Further combinatorial analysis of protein and gene expression in tumor tissue and peripheral CD8^+^ T cells revealed that the plasma concentrations of sPD-1, sPD-L1, and sCTLA-4 reflected the extent of exhaustion or overactivation of antitumor immunity in patients with advanced NSCLC. We believe that our study provides new insight into the role of soluble immune factors with regard to the relationship between immune exhaustion and clinical outcome for PD-1/PD-L1 blockade therapy.

## Results

### Characteristics of a prospective clinical trial cohort.

**Figure 1:**
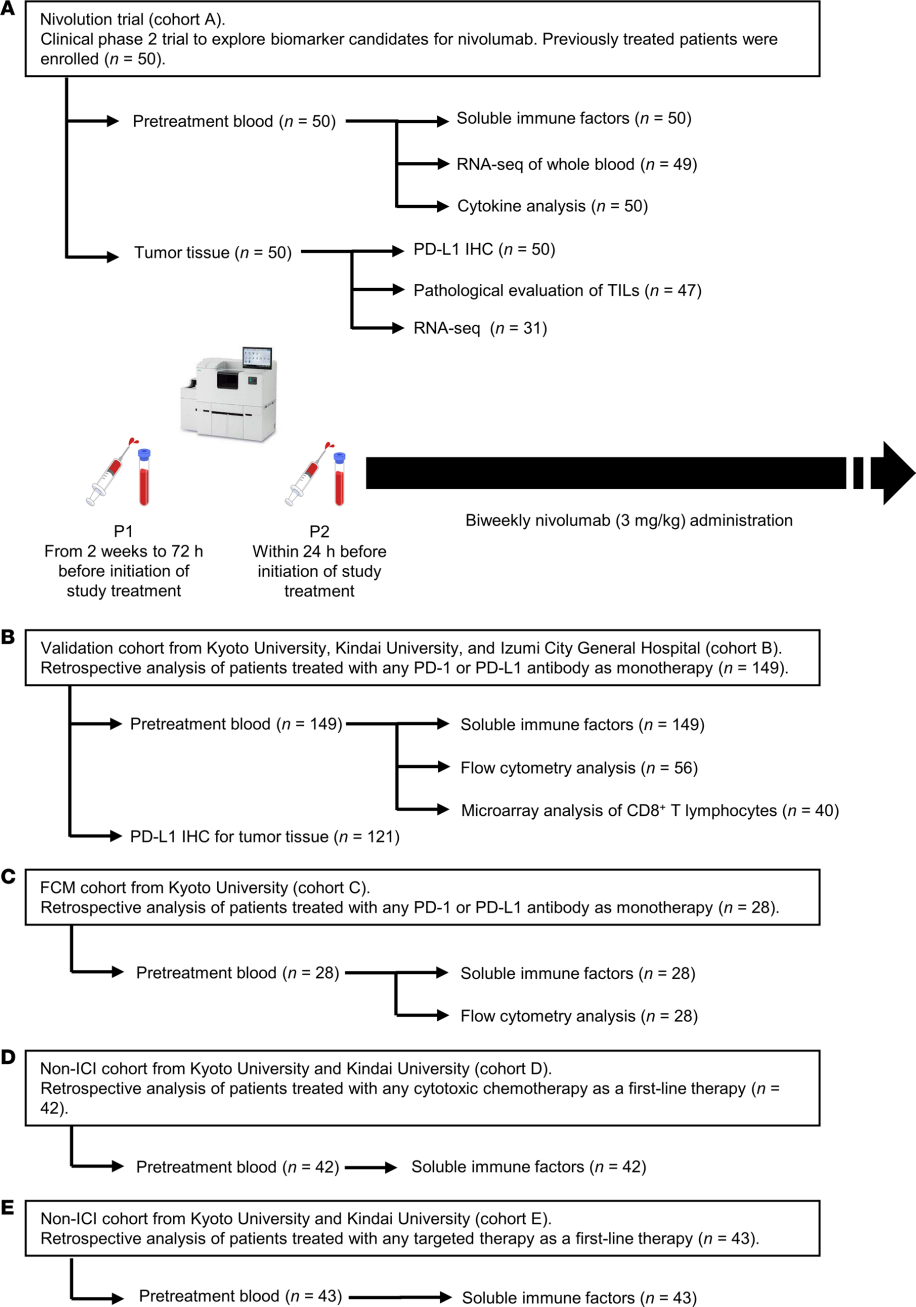
Overview of patient cohorts and analyses included in the study. (**A**) In a prospective trial to identify biomarker candidates for nivolumab treatment (Nivolution trial), a total 50 patients with advanced or recurrent NSCLC previously treated with any systemic therapy (cohort A) was analyzed. (**B**) Retrospective analysis of 149 patients with advanced or recurrent NSCLC who received monotherapy with any PD-1 or PD-L1 inhibitor in the first- or later-line setting (cohort B). Flow cytometry (FCM) and microarray analysis of gene expression were performed for peripheral CD8^+^ T cells from 56 and 40 patients, respectively, enrolled at Kyoto University Hospital, which was previously reported (27) (**C**) Retrospective analysis of patients who underwent more than 1 line of systemic therapy before ICI treatment at Kyoto University Hospital (cohort C) (27) (**D** and **E**) Retrospective analysis of 42 and 43 patients with advanced or recurrent NSCLC who received cytotoxic chemotherapy (cohort D) or targeted therapy (cohort E) in the first-line setting, respectively.

To investigate the potential of sPD-L1, sPD-1, and sCTLA-4 as biomarkers for ICI therapy, we examined 2 cohorts for discovery (Nivolution trial, cohort A) and validation (cohort B) (Figure 1, A and B). For the purpose of flow cytometric analysis, we examined cohort C, another retrospective validation cohort, in addition to cohort B (Figure 1C). In the prospective Nivolution trial, 50 patients with advanced NSCLC were enrolled and treated with nivolumab at a dose of 3 mg/kg biweekly (Figure 1A). For examination of the prediction of nivolumab efficacy by sPD-L1, sPD-1, and sCTLA-4 concentrations, plasma samples were collected from all 50 enrolled patients. The characteristics of the patients are shown in Table 1. At a median follow-up time of 13.2 months (range, 2.1–19.5 months), the median PFS and median overall survival were 3.6 months (95% CI, 2.2–9.1 months) and 15.2 months (95% CI, 13.2 months–not reached), respectively (Supplemental Figure 1, A and B; supplemental material available online with this article; https://doi.org/10.1172/JCI168318DS1). The overall response [complete response (CR) + partial response (PR)] rate and disease control [CR + PR + stable disease (SD)] rate were 24.0% (95% CI, 12.2–35.8%) and 58.0% (95% CI, 44.3–71.7%), respectively. The patient background and efficacy of nivolumab were consistent with the findings of previous trials (37, 38).

Pathological determination of tPD-L1 expression was performed for all 50 patients, with most of the tumor samples being obtained within 1 year of enrollment. Thirteen patients with high tPD-L1 expression [tumor proportion score (TPS) of ≥ 50%] showed superior nivolumab efficacy in terms of PFS compared with the remaining 37 patients with low tPD-L1 expression (TPS of < 50%) (Supplemental Figure 1C). In contrast, with a tPD-L1 of 1% as the cutoff, there was no clear difference in PFS for nivolumab between patients with a value of < 1% or ≥ 1% (Supplemental Figure 1D).

### Relation between sPD-L1, sPD-1, and sCTLA-4 levels in plasma before treatment and innate resistance to nivolumab.

Given that the concentrations of sPD-L1, sPD-1, or sCTLA-4 in 2 samples collected at 2 different time points from the same patients before treatment (P1 and P2 time points in Figure 1A) were consistent and showed minimal variability (Supplemental Figure 2, A–C), the plasma samples obtained at the time point closest to initiation of nivolumab treatment (P2) were subjected to further analysis. Triplicate assay of sPD-L1, sPD-1, and sCTLA-4 concentrations with the HISCL system was performed for all 50 patients, yielding median values of 217, 137, and 1.70 pg/mL, respectively (Supplemental Figure 3A). There were no significant differences in the levels of the 3 soluble factors with regard to sex, smoking history (never versus former or current), histology (nonsquamous versus squamous), *EGFR* or *ALK* gene alterations (WT versus altered in nonsquamous lung carcinoma), and number of prior chemotherapy lines (1 versus ≥ 2), with the exception of sPD-L1 concentration and sex (Supplemental Figure 3, B–F). The concentration of sCTLA-4 was moderately correlated with that of sPD-1, whereas those of sPD-L1 and sPD-1 were not correlated (Supplemental Figure 4, A–C).

**Figure 2:**
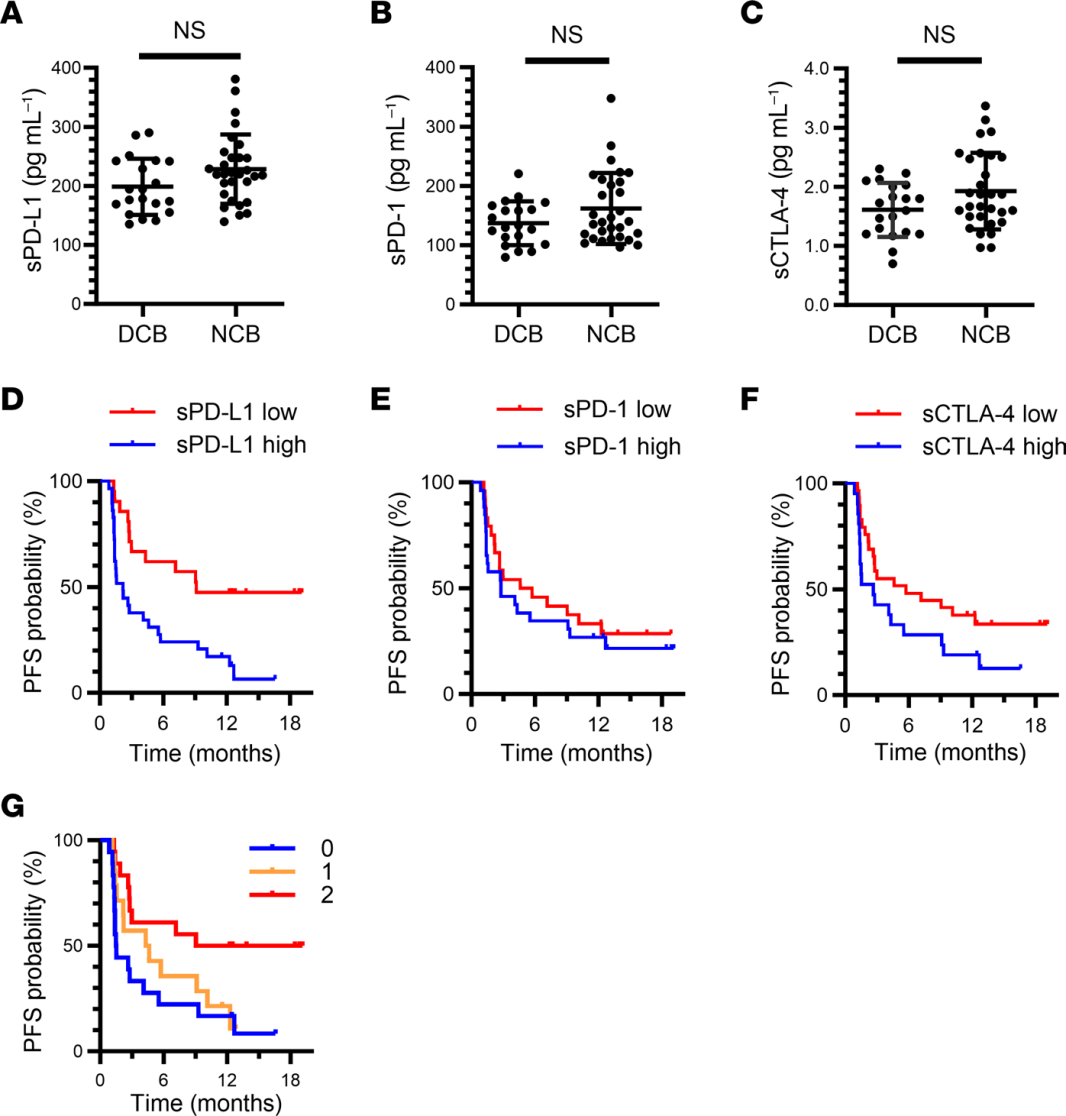
Combination of circulating soluble immune factors allows stratification of patients with advanced NSCLC in the Nivolution trial according to responsiveness to nivolumab. (**A**–**C**) Comparison of pretreatment plasma concentrations of sPD-L1 (**A**), sPD-1 (**B**), and sCTLA-4 (**C**) between patients with a DCB (*n* = 20) or NCB (*n* = 30). Mean ± SD values are indicated; Mann-Whitney U test. (**D**–**F**) Kaplan-Meier curves for PFS of patients with high or low concentrations of each soluble immune factor based on the determined cutoff values. For **D**, the sPD-L1 cutoff was 205 pg/mL (high, *n* = 29; low, *n* = 21), and the median PFS was 9.1 versus 2.2 months for low and high sPD-L1, respectively (log-rank *P* = 0.002), with an HR of 0.35 (95% CI, 0.18–0.68). For **E**, the sPD-1 cutoff was 135 pg/mL (high, *n* = 26; low, *n* = 24), and the median PFS was 5.2 versus 2.8 months for low and high sPD-1, respectively (log-rank *P* = 0.459), with an HR of 0.78 (95% CI, 0.41–1.50). For **F**, the sCTLA-4 cutoff was 1.85 pg/mL (high, *n* = 21; low, *n* = 29), and the median PFS was 5.7 versus 2.7 months for low and high sCTLA-4, respectively (log-rank *P* = 0.074), with an HR of 0.54 (95% CI, 0.27–1.06). (**G**) Kaplan-Meier curves for PFS among patients according to the number of favorable immune factors defined as sCTLA-4 or sPD-L1 levels below the cutoff values (log-rank *P* = 0.015). Median PFS was 14.1, 4.5, and 1.5 months for 2, 1, and 0 favorable factors, respectively. The HR for 1 (*n* = 14) versus 0 (*n* = 18) was 0.72 (95% CI, 0.34–1.53), and that for 2 (*n* = 18) versus 0 was 0.31 (95% CI, 0.14–0.72).

The pretreatment plasma concentrations of sPD-L1, sPD-1, and sCTLA-4 tended to be lower in patients with a durable clinical benefit (DCB: CR, PR, or SD lasting > 6 months) than in those with no clinical benefit (NCB) of nivolumab treatment (Figure 2, A–C). Each single soluble factor was moderately predictive for the 6-month PFS rate, with an area under the receiver operator characteristic (ROC) curve of 0.64, 0.60, and 0.63 for sPD-L1, sPD-1, and sCTLA-4, respectively (Supplemental Figure 5, A–C). We determined the cutoff values for such prediction as 205, 135, and 1.85 pg/mL for sPD-L1, sPD-1, and sCTLA-4, respectively, by combining high sensitivity and best specificity in the ROC curves for 6-month PFS rate with the use of a proportional hazards model (Supplemental Methods). A Venn diagram for patients with high levels of the soluble immune factors is shown in Supplemental Figure 6A. Patients with values below these cutoff points showed a longer PFS than did those with values above them, although the difference was minor for sPD-1 (Figure 2, D–F). Overall, the efficacy of nivolumab tended to be poor in patients with a high concentration of sPD-L1, sPD-1, or sCTLA-4.

We further investigated whether a combination of these soluble immune factors might improve their predictive ability relative to each marker alone for PD-1/PD-L1 blockade therapy Given that sPD-L1 and sCTLA-4 showed a higher sensitivity than did sPD-1 (Figure 2, D–F), we combined these 2 factors for the analysis. We defined the presence of sPD-L1 or sCTLA-4 at a concentration below the cutoff value as a favorable factor. The 6-month PFS rate and median PFS of the group with 2 favorable factors were 61.1% and 14.1 months, respectively, whereas those for the group with a single favorable factor were 35.7% and 4.5 months and those for the group with no favorable factors were 22.2% and 1.5 months (Figure 2G). These findings suggested that the combination of soluble immune factors improved prediction of the therapeutic efficacy of nivolumab.

### Soluble immune factors complement patient stratification by tPD-L1.

Given that tPD-L1 expression is the most well-established biomarker for PD-1 blockade cancer immunotherapy in patients with advanced NSCLC (Supplemental Figure 1, C and D) (39), we next investigated the relationship between tPD-L1 and the 3 soluble immune markers.

**Figure 3:**
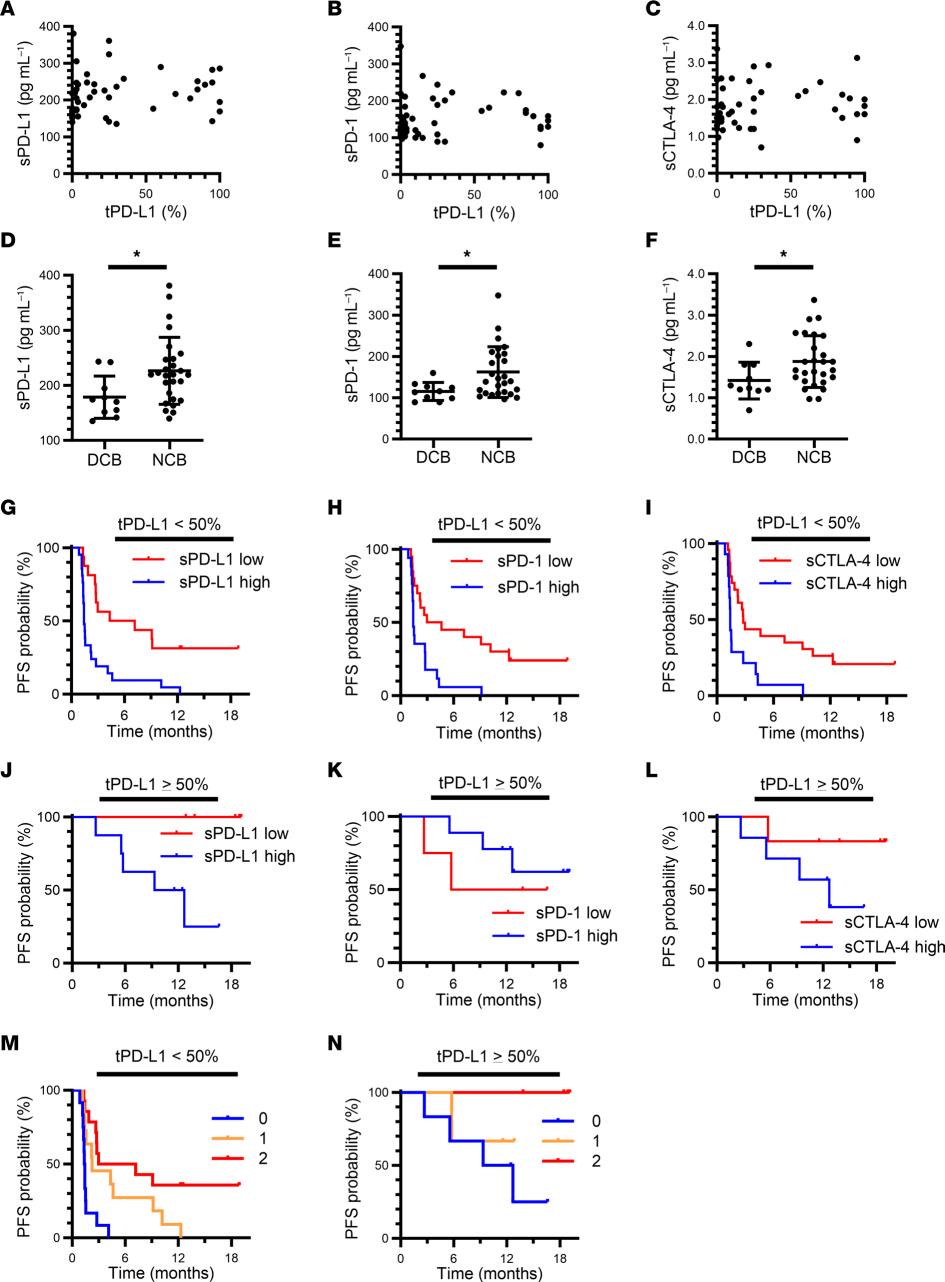
Soluble immune factors complement the predictive ability of tPD-L1 expression for advanced NSCLC patients treated with nivolumab in the Nivolution trial. (**A**–**C**) Pearson correlation analysis for pretreatment plasma concentrations of sPD-L1 (**A**), sPD-1 (**B**), sCTLA-4 (**C**), and tPD-L1 expression level (PD-L1 TPS) (*n* = 50). (**D**–**F**) Comparison of sPD-L1 (**D**), sPD-1 (**E**), and sCTLA-4 (**F**) concentrations between patients with a DCB (*n* = 10) or NCB (*n* = 27) among individuals with a tPD-L1 expression level of < 50%. *P < 0.05 (Mann-Whitney U test). (**G**–**I**) Kaplan-Meier curves for PFS of patients with a tPD-L1 expression level of < 50% according to high or low levels of each soluble immune factor based on the determined cutoff values. For sPD-L1 (high, *n* = 21; low, *n* = 16), median PFS was 8.7 versus 2.7 months for low and high sPD-L1, respectively (log-rank *P* = 0.001), with an HR of 0.30 (95% CI, 0.14–0.64) (**G**). For sPD-1 (high, *n* = 20; low, *n* = 17), median PFS was 7.8 versus 2.4 months for low and high sPD-1, respectively (log-rank *P* = 0.003), with an HR of 0.34 (95% CI, 0.16–0.71) (**H**). For sCTLA-4 (high, *n* = 14; low, *n* = 23), median PFS was 7.1 versus 2.4 months for low and high sCTLA-4, respectively (log-rank *P* = 0.004), with an HR of 0.36 (95% CI, 0.17–0.75) (**I**). (**J**–**L**) Kaplan-Meier curves for PFS of patients with a tPD-L1 expression level of ≥ 50% according to high or low levels of each soluble immune factor based on the determined cutoff values. For sPD-L1 (high, *n* = 8; low, *n* = 5), median PFS was not reached versus 11.0 months for low and high sPD-L1, respectively (log-rank *P* = 0.023), with an HR of 0.01 (95% CI, 0.00–19.61) (**J**). For sPD-1 (high, *n* = 8; low, *n* = 5), median PFS was 5.7 months versus not reached for low and high sPD-1, respectively (log-rank *P* = 0.49), with an HR of 1.88 (95% CI, 0.31–11.32) (**K**). For sCTLA-4 (high, *n* = 7; low, *n* = 6), median PFS was not reached versus 12.7 months for low and high sCTLA-4, respectively (log-rank *P* = 0.16), with an HR of 0.23 (95% CI, 0.03–2.14) (**L**). (**M** and **N**) Kaplan-Meier curves for PFS among patients with tPD-L1 expression levels of < 50% (**M**) or ≥ 50% (**N**) according to the number of favorable immune factors defined as sCTLA-4 or sPD-L1 concentrations below the cutoff values (log-rank *P* = 0.0002 and 0.18, respectively). Median PFS was 5.1, 2.2, and 1.4 months for 2, 1, and 0 favorable factors, respectively, in (**M**), and not reached, not reached, and 11.0 months, respectively, in (**N**). The HR for 1 (*n* = 11 and 3) versus 0 (*n* = 12 and 6) was 0.28 (95% CI, 0.10–0.76) and 0.44 (95% CI, 0.05–3.97), and that for 2 (*n* = 14 and 4) versus 0 was 0.20 (95% CI, 0.10–0.76) and 0.01 (95% CI, 0.00–45.45), in (**M**) and (**N**), respectively.

None of the soluble markers showed a clear correlation with tPD-L1 (Figure 3, A–C). For patients with a low tPD-L1 expression (TPS of < 50%), the pretreatment level of sPD-L1, sPD-1, or sCTLA-4 was significantly higher in patients with NCB than in those with a DCB (Figure 3, D–F). Furthermore, the cutoff values determined above for each soluble immune factor appeared to be more discriminative in patients with a low tPD-L1 level (Figure 3, G–I) than in all patients (Figure 2, D–F). For patients with high tPD-L1 expression (TPS of ≥ 50%), those with high sPD-L1 or sCTLA-4 concentrations were also more resistant to nivolumab treatment than were those with low concentrations, although the difference was statistically significant only for sPD-L1, possibly as a result of the small number of cases (Figure 3, J–L). The baseline levels of sPD-L1, sPD-1, and sCTLA-4 for these patients according to treatment response are shown in Supplemental Figure 7.

We then conducted an integrative analysis for sPD-L1 and sCTLA-4 levels according to tPD-L1 status. A greater predictive ability was apparent in the low tPD-L1 group (Figure 3M) than in all patients (Figure 2G). Despite the limited number of patients, a similar predictive tendency was observed in the high tPD-L1 group (Figure 3N). For the cohorts defined by a tPD-L1 cutoff of 1%, sPD-L1 and sCTLA-4 levels stratified the patients with a similar trend (Supplemental Figure 8, A and B). Together, these results suggested that the combination of sPD-L1 and sCTLA-4 levels showed improved prediction of nivolumab efficacy, even for patients stratified by tPD-L1 expression, thus complementing the predictive ability of tPD-L1.

### The combination of sPD-L1 and sCTLA-4 predicts nivolumab efficacy in patients with high tPD-L1 expression.

**Figure 4:**
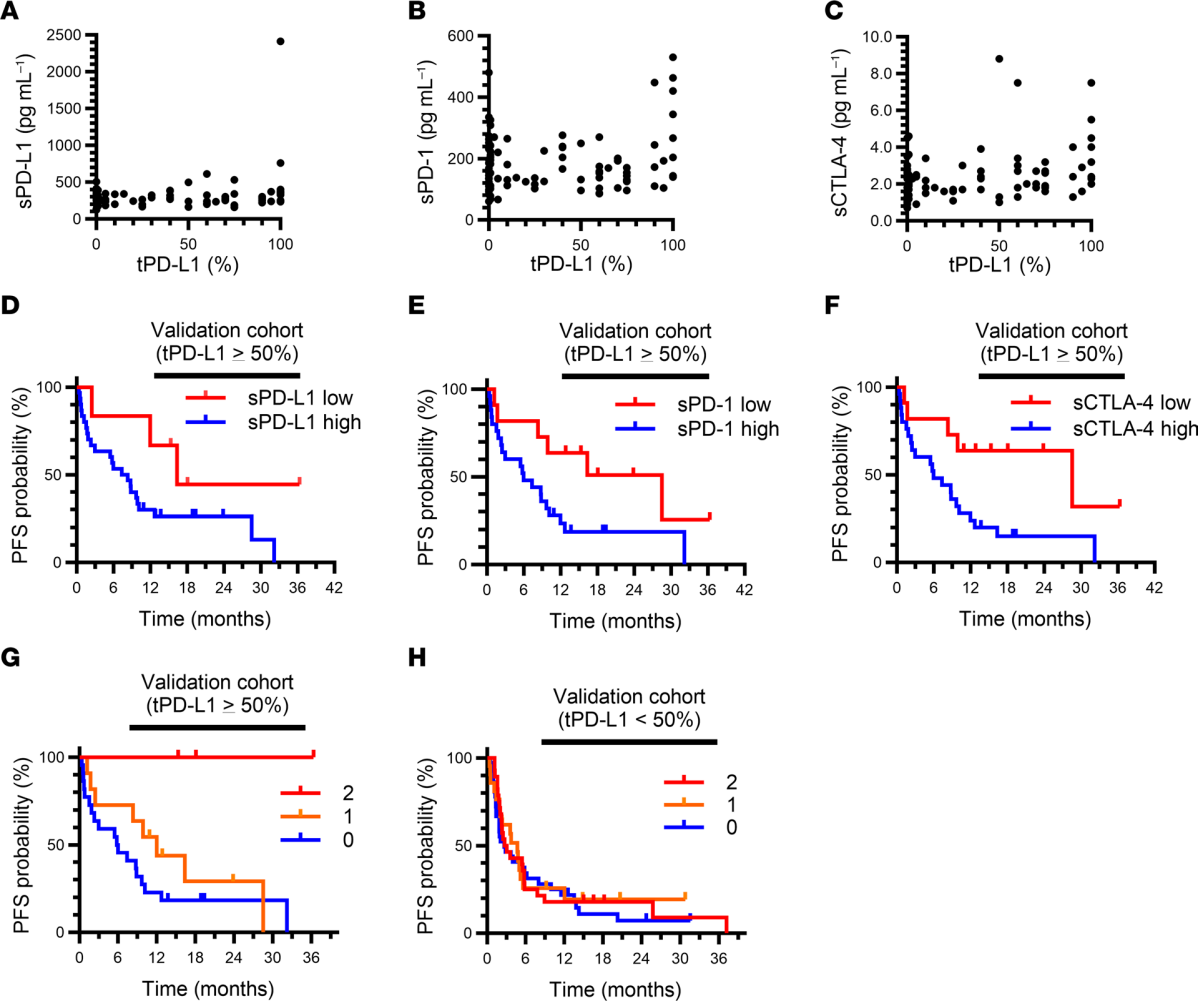
Soluble immune factors stratify advanced NSCLC patients with a tPD-L1 expression level of ≥ 50% according to responsiveness to PD-1/PD-L1 blockade therapy in the validation cohort (cohort B). (**A**–**C**) Pearson correlation analysis of pretreatment plasma concentrations of sPD-L1 (**A**), sPD-1 (**B**), or sCTLA-4 (**C**) and tPD-L1 expression level (*n* = 121 patients). (**D**–**F**) Kaplan-Meier curves for PFS of patients with a tPD-L1 expression level of ≥ 50% according to high or low levels of soluble immune factors based on the determined cutoff values. For sPD-L1 (high, *n* = 30; low, *n* = 6), median PFS was 16.4 versus 7.4 months for low and high sPD-L1, respectively (log-rank *P* = 0.080), with an HR of 0.35 (95% CI, 0.11–1.19) (**D**). For sPD-1 (high, *n* = 25; low, *n* = 11), median PFS was 28.6 versus 6.0 months for low and high sPD-1, respectively (log-rank *P* = 0.035), with an HR of 0.38 (95% CI, 0.15–0.97) (**E**). For sCTLA-4 (high, *n* = 25; low, *n* = 11), median PFS was 28.6 versus 6.0 months for low and high sCTLA-4, respectively (log-rank *P* = 0.017), with an HR of 0.32 (95% CI, 0.12–0.86) (**F**). (**G** and **H**) Kaplan-Meier curves for PFS among patients with a tPD-L1 expression level of ≥ 50% (**G**) or < 50% (**H**) according to the number of favorable immune factors defined as concentrations of sCTLA-4 or sPD-L1 below the cutoff values (log-rank *P* = 0.028 and 0.57, respectively). Median PFS was not reached, 11.0 months, and 5.9 months for 2, 1, and 0 favorable factors, respectively, in **G**, and 2.9, 4.7, and 2.7 months, respectively, in **H**. The HR for 1 (*n* = 11 and 21) versus 0 (*n* = 22 and 36) was 0.61 (95% CI, 0.26–1.41) and 0.84 (95% CI, 0.46–1.54), and that for 2 (*n* = 3 and 28) versus 0 was 0.03 (95% CI, 0.00–3.43) and 0.88 (95% CI, 0.52–1.50), in (**G**) and (**H**), respectively.

To validate the predictive value of soluble immune factors revealed by our analysis of discovery cohort A, we performed a retrospective analysis of another cohort (cohort B) of ICI-treated patients with advanced NSCLC (Figure 1B). This cohort included 149 patients with NSCLC who had been treated with any antibody to PD-1 or to PD-L1 at Kyoto University, Kindai University, or Izumi City General Hospital (Table 1). Soluble immune factors were measured in plasma samples of all 149 patients, and data for PD-L1 expression on tumor cells were available for 121 patients. For this cohort, the soluble factor concentrations were weakly or not correlated with tPD-L1 expression (Figure 4, A–C). A Venn diagram for patients with high levels of the soluble immune factors in cohort B is shown in Supplemental Figure 6B. Whereas there was no significant difference in PFS between patients with high or low levels of sPD-L1, sPD-1, or sCTLA-4 (according to the predefined cutoff values) in the group with low tPD-L1 expression (TPS of < 50%) (Supplemental Figure 9, A–F), patients with high concentrations of sPD-L1, sPD-1, or sCTLA-4 were more resistant to ICI treatment than were those with low concentrations in the group with high tPD-L1 expression (TPS of ≥ 50%) (Figure 4, D–F). Similar to the discovery cohort, combination analysis of sPD-L1 and sCTLA-4 revealed that patients with low sPD-L1 and sCTLA-4 levels showed a longer PFS in the high tPD-L1 group (Figure 4G). However, there was no such significant difference apparent for the low tPD-L1 group (Figure 4H). The findings from both cohorts A and B together revealed that the combination of sPD-L1 and sCTLA-4 is potentially effective as a biomarker for PD-1/PD-L1 blockade therapy, especially for patients with high tPD-L1 expression.

To confirm that the predictive efficacy of soluble immune factors is specific to ICIs, we conducted a separate analysis of additional cohorts to investigate the relation between soluble immune factors and the effectiveness of cytotoxic chemotherapy (cohort D) or tyrosine kinase inhibitors (TKIs) (cohort E) (Figure 1, D and E, and Supplemental Table 1). There was no significant difference in PFS between high and low levels of sPD-L1, sPD-1, or sCTLA-4, or according to the combination of sPD-L1 and sCTLA-4 at concentrations below the cutoff values, for patients treated with chemotherapy (Supplemental Figure 10). Similar results were obtained for the patients treated with TKIs (Supplemental Figure 11). These findings thus supported the specific predictive ability of the combination of sPD-L1 and sCTLA-4 levels for the effectiveness of PD-1/PD-L1 blockade therapy.

### Low concentrations of soluble immune factors better predict responders among patients with immune-reactive tumors.

T cell–mediated antitumor immune responses are important for the effective destruction of malignant cells during ICI therapy. Tumors with a high frequency of CD8^+^ T cell infiltration are more likely to be immune-reactive, or “hot” (40–42). To examine whether sPD-L1, sPD-1, and sCTLA-4 levels might be dependent on T cell–mediated immune reactions to cancer, we explored their relation to the number of CD8^+^ tumor-infiltrating lymphocytes (TILs) in cohort A.

**Figure 5:**
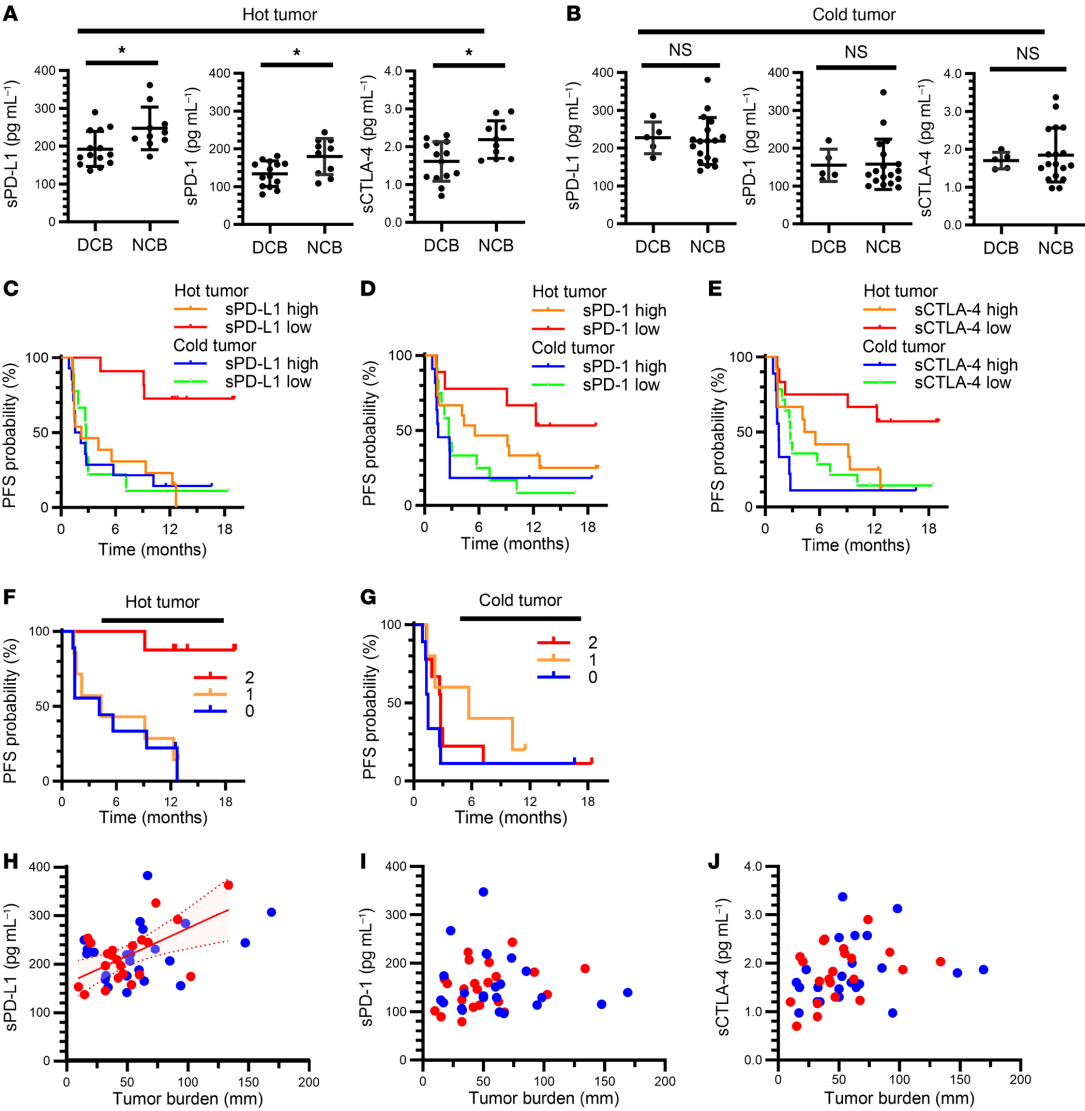
Soluble immune factors efficiently stratify patients with hot tumors in the Nivolution trial. (**A** and **B**) Comparison of pretreatment plasma concentrations of sPD-L1, sPD-1, or sCTLA-4 between patients with a DCB or NCB among individuals with hot (**A**) or cold (**B**) tumors defined by the number of CD8^+^ T cells infiltrated into tumor tissue (≥ 12.0 and < 12.0/field, respectively). DCB, *n* = 14 and 5; NCB, *n* = 10 and 18 for hot and cold tumors, respectively. *P < 0.05; Mann-Whitney U test. (**C**–**E**) Kaplan-Meier curves for PFS according to hot or cold tumor status and high or low soluble factor levels based on the determined cutoff values. For sPD-L1, 2-sided log-rank *P* = 0.0023 for comparison among the 4 groups, where *n* = 11 (sPD-L1 low) and 13 (sPD-L1 high) among hot tumors as well as *n* = 9 (low) and 14 (high) among cold tumors; and median PFS was not reached, 2.2 months, 2.8 months, and 1.9 months, respectively (**C**). For sPD-1, 2-sided log-rank *P* = 0.055; *n* = 9 (sPD-1 low) and 15 (sPD-1 high) among hot tumors as well as *n* = 12 (low) and 11 (high) among cold tumors; and median PFS was not reached, 5.6 months, 2.7 months, and 1.5 months, respectively (**D**). For sCTLA-4, 2-sided log-rank *P* = 0.0093; *n* = 12 (sCTLA-4 low) and 12 (sCTLA-4 high) among hot tumors as well as *n* = 14 (low) and 9 (high) among cold tumors; and median PFS was not reached, 4.9 months, 2.8 months, and 1.5 months, respectively (**E**). (**F** and **G**) Kaplan-Meier curves for PFS of patients with hot (**F**) or cold (**G**) tumors according to the number of favorable immune factors defined as concentrations of sCTLA-4 or sPD-L1 below the cutoff values (2-sided log-rank *P* = 0.0034 and 0.30, respectively). Median PFS was not reached, 4.3 months, and 4.1 months for 2, 1, and 0 favorable factors, respectively, for hot tumors (**F**), and was 2.8, 5.7, and 1.5 months, respectively, for cold tumors (**G**). The HR for 1 (*n* = 7 and 5) versus 0 (*n* = 9 and 9) was 0.85 (95% CI, 0.29–2.44) and 0.46 (95% CI, 0.13–1.57), and that for 2 (*n* = 8 and 9) versus 0 was 0.07 (95% CI, 0.01–0.55) and 0.58 (95% CI, 0.21–1.56), for hot and cold tumors, respectively. (**H**–**J**) Pearson correlation analysis of tumor burden and plasma concentrations of sPD-L1 (**H**), sPD-1 (**I**), or sCTLA-4 (**J**) for hot (red) and cold (blue) tumors. Hot tumors in **H** show moderate linearity, with an *R* of 0.59 and *P* = 0.004. The red shaded area above and below the solid line and bounded by the dotted lines indicates the 95% CI.

Consistent with previous findings (41–43), pathological examination revealed that patients with a high frequency of CD8^+^ T cells in tumor tissue (hot tumors: ≥ 12.0/field) showed a better PFS during nivolumab treatment compared with those with a low frequency (cold tumors: < 12.0/field) (Supplemental Figure 12A). The CD8^+^ TIL density was not correlated with baseline concentrations of the soluble immune factors among the patients (*n* = 47) subjected to the pathological analysis (Supplemental Figure 12, B–D). However, for the hot tumor group, the concentrations of these soluble factors were significantly higher in patients with NCB than in those with a DCB (Figure 5A), whereas no such significant difference was apparent for the cold tumor group (Figure 5B). Combination of the cutoff values for the soluble factors and the hot and cold tumor classification revealed that patients with a low concentration of sPD-L1, sPD-1, or sCTLA-4 and a hot tumor had the best PFS compared with the other combined groups (Figure 5, C–E). Although high tPD-L1 expression has been thought to result from an immune reaction mediated by IFN-γ released from CD8^+^ T cells, we found that CD8^+^ TIL density did not show a clear association with tPD-L1 expression (Supplemental Figure 12E). Given that the combination of sPD-L1 and sCTLA-4 effectively discriminated responsiveness in patients with high tPD-L1 expression (TPS of ≥ 50%) (Figure 3N and Figure 4G), we next examined whether this combination might also be discriminative for hot tumors. The combination of low sPD-L1 and low sCTLA-4 was associated with a better PFS for hot tumors, but not for cold tumors (Figure 5, F and G). Together, our results suggested that lower plasma concentrations of the soluble immune factors were more likely to distinguish responders from nonresponders among patients with hot tumors than among those with cold tumors, indicating that these soluble factors are more meaningful in patients with immune-reactive tumors.

### Soluble immune factors show a better correlation with tumor burden in patients with hot tumors.

Recent single-cell analysis technology has revealed that PD-1 blockade therapy results in replacement of CD8^+^ T cell clones at tumor sites through depletion of terminally exhausted T cells and expansion of progenitor exhausted T cells in responsive patients (34, 35). This finding suggests that progenitor exhausted tumor-reactive T cells are important for a response, and that antitumor immunity that has already been excessively activated cannot be rejuvenated further by PD-1 blockade (25, 28, 33). Markers reflecting an exhausted (overactivated) state of T cells might therefore serve as negative indicators for the efficacy of PD-1 blockade therapy (26, 27). On the basis of our results showing that sPD-L1, sPD-1, and sCTLA-4 are most relevant for patients with tumors that show a high level of PD-L1 expression and those with immune-reactive tumors characterized by high CD8^+^ TIL infiltration and that patients with high levels of the soluble immune factors tend to be unresponsive, we hypothesized that these soluble factors might reflect the systemic level of overactivated or exhausted T cells.

To test this hypothesis, we compared exhaustion status and soluble immune factors in several ways. We and others previously showed that a large tumor size is often associated with a poor outcome of ICI therapy in patients with advanced NSCLC (26, 44). In such patients, the number of hyperactivated or exhausted T cells was positively correlated with tumor burden (26). Indeed, in cohort A of the present study, PFS for nivolumab was significantly longer in patients with smaller tumors than in those with a larger tumor burden (Supplemental Figure 13). We therefore investigated whether the soluble immune markers were related to tumor burden. Whereas the plasma concentrations of sPD-L1, sPD-1, and sCTLA-4 did not show a clear linear correlation with baseline tumor size among patients in cohort A overall (Supplemental Figure 14, A–C), a relation was observed between sPD-L1 and tumor burden for patients with hot tumors but not for those with cold tumors (Figure 5, H–J). Of note, multivariate analysis in cohort A showed that only tPD-L1 and favorable immune factors, not tumor burden, were predictive of nivolumab efficacy (Supplemental Table 2). These data suggested that, in patients with antitumor T cell responses within the tumor, the plasma levels of the soluble immune factors might reflect the hyperactivation or exhaustion of T cells.

### Soluble immune factors are related to exhaustion status of antitumor immunity.

**Figure 6:**
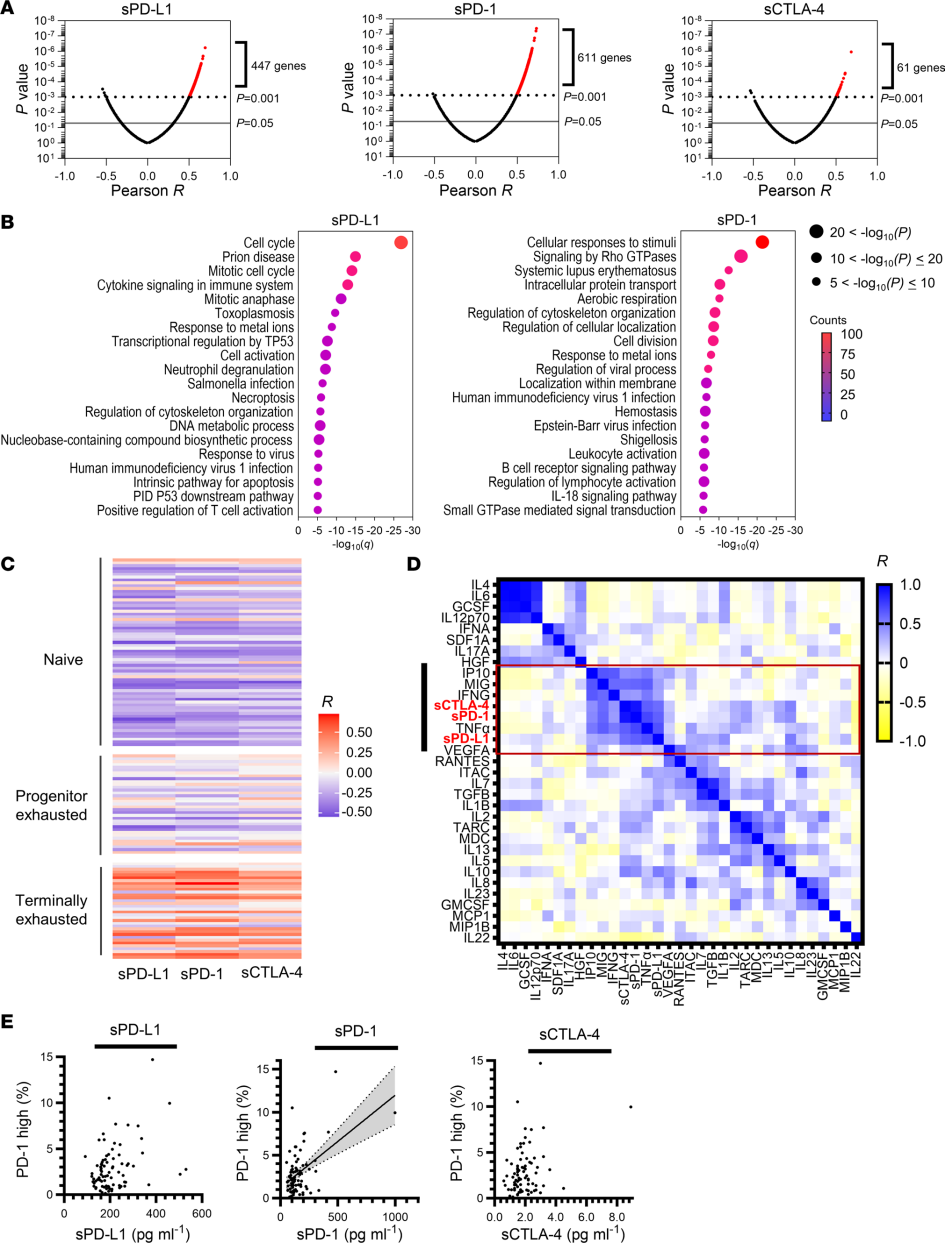
Analysis of gene expression in peripheral CD8^+^ T cells and circulating cytokine levels. (**A**) Volcano plots of Pearson correlation (*x*-axis) and significance (*y*-axis) for the expression of individual genes in peripheral CD8^+^ T cells as determined by microarray analysis and pretreatment plasma concentrations of sPD-L1, sPD-1, or sCTLA-4 in cohort B (validation cohort, *n* = 40 patients). (**B**) Enrichment analysis for genes whose expression was positively correlated with sPD-L1 (447 genes) or sPD-1 (611 genes) levels as shown in **A** at *P* values of < 0.001. The plots show the FDR (*q*) value (*x*-axis), adjusted *P* value (dot size), and gene counts (color). The number of correlated genes for sCTLA-4 was not sufficient for enrichment analysis. (**C**) Heat map of Pearson correlation between soluble immune factor concentrations and the expression of gene sets characteristic of naive, progenitor exhausted, or terminally exhausted CD8^+^ T cells as determined by microarray analysis as in **A**. (**D**) Correlation between the plasma concentrations of 30 cytokines as well as those of sPD-L1, sPD-1, and sCTLA-4 in 50 patients of cohort A (Nivolution trial). Hierarchical clustering was performed according to the concentrations of the cytokines and soluble immune factors. (**E**) Scatter plots of soluble immune factor levels and the frequency of PD-1^hi^ CD8^+^ T cells in peripheral blood (*n* = 84 from cohorts B and C). A moderate correlation between sPD-1 levels and the frequency of PD-1^hi^ CD8^+^ T cells was apparent, with an *R* value of 0.51 and *P* < 0.0001; the gray shaded area above and below the solid line and bounded by the dotted lines indicates the 95% CI.

To further investigate whether higher concentrations of sPD-L1, sPD-1, and sCTLA-4 reflect the exhaustion status of T cell–mediated antitumor immunity, we next examined the relation between gene expression in peripheral CD8^+^ T cells and the concentration of each soluble immune factor with the use of pretreated blood samples from 40 patients with NSCLC from cohort B (Figure 1B) (27). We found that the expression of a substantial number of genes was positively correlated with the concentrations of the soluble immune factors (Figure 6A and Supplemental Table 3). With the exception of sCTLA-4, the number of correlated genes was sufficient for pathway analysis (Figure 6B). The enriched pathways for genes whose expression was positively correlated with sPD-L1 concentration included those related to cell proliferation and immune activation, whereas enriched pathways for genes correlated with sPD-1 level included those related to immune responses, T cell activation, and T cell anergy (signaling by Rho GTPases; Figure 6B) (45). Examination of genes characteristic of naive, progenitor exhausted, or terminally exhausted T cells (46) revealed that the concentration of each soluble immune factor was highly correlated with the expression of genes associated with terminally exhausted T cells, but not with that of those associated with naive or progenitor exhausted phenotypes (Figure 6C and Supplemental Table 4). Consistent with these findings, examination of the relation between the plasma concentrations of 30 cytokines and those of sPD-L1, sPD-1, and sCTLA-4 in cohort A revealed that IFN-γ and IFN-γ–induced chemokines were strongly or moderately correlated with the 3 soluble markers (Figure 6D). These data thus supported the hypothesis that the 3 soluble factors reflected the extent of exhaustion of antitumor immunity. Whereas the plasma concentrations of sPD-L1, sPD-1, and sCTLA-4 were not correlated with expression of the corresponding genes (*CD274*, *PDCD1*, and *CTLA4*, respectively) in tumor specimens (Supplemental Figure 15, A–C), that of sPD-L1 was moderately correlated with the expression of *CD274* in whole-blood cells (Supplemental Figure 15D), suggesting that sPD-L1 might be preferentially derived from blood cells rather than tumor tissue, possibly as a result of the induction of peripheral PD-L1 expression by circulating IFN-γ (Figure 6D). It is of note that the frequency of PD-1^hi^ CD8^+^ T cells, which are thought to be terminally exhausted (27), among PBMCs of patients in cohorts B and C (*n* = 84) was moderately correlated with plasma sPD-1 concentration (Figure 6E), indicating that sPD-1 is derived in part from terminally exhausted CD8^+^ T cells.

## Discussion

Our results indicate that the plasma concentrations of soluble checkpoint molecules, especially the combination of sPD-L1 and sCTLA-4, can serve as a complementary predictive factor in patients with a high level of tPD-L1 expression. The combination of PD-1/PD-L1 inhibitors and cytotoxic chemotherapy is currently still recognized as a standard treatment even for patients with high tPD-L1 expression not subjected to selection based on a clear prescription biomarker (47). Although further study is needed to determine the relationship of soluble immune factors to the efficacy of the combination of chemotherapy and ICI therapy, these factors might be able to stratify even patients considered for such treatment. Furthermore, the collection of tumor tissue in a timely manner can be challenging and may not accurately reflect the actual circumstances at the time of analysis. In contrast, liquid biopsy offers a simple and minimally invasive approach, providing a highly informative means to assess the immune environment in real time. Analysis of the soluble immune factors examined here has the potential to serve as an alternative to analysis of tumor tissue for evaluation of the immune landscape, and examination of the effectiveness of ICIs in patient subgroups with a tPD-L1 of ≥ 50% and with low sPD-L1 and sCTLA-4 levels in a future prospective study is warranted.

Recent analysis by single-cell sequencing technology of clinical samples obtained before and after PD-1 blockade has suggested that such treatment boosts the proliferation of progenitor exhausted CD8^+^ T cells rather than rejuvenating exhausted cells (34, 35). In other words, an immune status characterized by the accumulation of overactivated or terminally exhausted T cells is unfavorable for cancer immunotherapy (25, 28, 33). Markers that reflect systemic T cell overactivation or terminal exhaustion might therefore serve as effective biomarkers for unresponsiveness to PD-1/PD-L1 blockade therapy. Given that PD-1 and CTLA-4 are expressed predominantly on activated or exhausted T cells (24–27) and that PD-L1 is highly expressed on various cell types according to immune reactions (15), it is reasonable that sPD-1, sCTLA-4, and sPD-L1 are associated with responsiveness in patients with immune-reactive (hot) tumors. The relationship of these soluble immune factors to activated or exhausted immune status is consistent with the finding that their circulating levels are higher in patients with cancer than in people from a healthy control group (36). In patients with advanced pancreatic cancer, the serum concentration of C-reactive protein (CRP), a marker of systemic inflammation, was found to be positively correlated with those of sPD-1 and sPD-L1 (48), also supporting our hypothesis.

We previously found that a high frequency of PD-1^hi^ CD8^+^ T cells in the periphery was able to discriminate unresponsiveness to PD-1 blockade therapy and that this cell population is highly exhausted (27). Our observation that the frequency of PD-1^hi^ CD8^+^ T cells in the periphery was moderately correlated with the plasma concentration of sPD-1 suggests that sPD-1 might be derived in part from peripheral exhausted CD8^+^ T cells positive for PD-1. With regard to the origin of sPD-L1, its plasma concentration was significantly correlated with *CD274* expression in whole blood, but was not correlated with CD274 expression in tumor tissue or with tPD-L1 expression, indicating that sPD-L1 in plasma might be derived predominantly from blood cells. Although the plasma level of sCTLA-4 was correlated with the expression of genes related to exhaustion in peripheral CD8^+^ T cells, fewer genes in these cells were significantly correlated with sCTLA-4 concentration than with sPD-L1 or sPD-1 concentration. Whereas plasma sCTLA-4 appeared to be correlated with activated immune status, it was likely derived from CD4^+^ T cells, including FOXP3^+^ T cells with high CTLA-4 expression (21), rather than from CD8^+^ T cells. Further investigation is necessary to identify where and from which cell populations these soluble immune factors are derived.

Our HISCL system allows the detection of low concentrations of soluble immune markers with high sensitivity and reproducibility (36). The circulating concentration of sCTLA-4 in particular is relatively low and difficult to detect by conventional ELISA kits, which has delayed studies of sCTLA-4 as a clinical marker. Although sPD-1, sPD-L1, and sCTLA-4 have been shown to be released from cells in a manner dependent on cell activation (7, 12, 19, 21), it remains a challenge to discriminate between the soluble forms derived from alternative RNA splicing and those produced by proteolytic shedding from the cell surface (8, 12, 20). The presence of sPD-L1–expressing exosomes in blood was recently found to be highly related to the efficacy of PD-1 blockade therapy (49). Our HISCL system detects various sPD-L1 isoforms including one derived from exosomes, but discrimination among these isoforms remains difficult.

In summary, our data suggest that sPD-1, sPD-L1, and sCTLA-4 in plasma reflect overactivation or exhaustion status of antitumor immunity. Although subset analysis in the current study is underpowered, and its results should therefore be interpreted with caution, these soluble immune factors serve as better biomarkers for PD-1/PD-L1 blockade therapy in patients with a high tPD-L1 expression or with immune-reactive (hot) tumors characterized by a high level of CD8^+^ T cell infiltration in tumor tissue. Indeed, the combination of sPD-L1 and sCTLA-4 was able to substantially stratify patients with advanced NSCLC with such tumors in our study and to complement the stratification ability of tPD-L1 expression that is currently in use. Our precise and reproducible system for measurement of sPD-L1 and sCTLA-4 in plasma would support the application of these biomarkers to stratify patients with advanced NSCLC in clinical practice.

## Methods

### Sex as a biological variable.

In this study, sex was not considered as a biological variable.

### Study design and patients.

The overall design of the study is outlined in Figure 1 and details of this section are described in Supplemental Methods. From December 2015 to September 2016, 50 previously treated patients with advanced or recurrent NSCLC were prospectively enrolled in a Phase II biomarker-finding trial, Nivolution, that was conducted at Kindai University Hospital.

For the cohorts B and C, patients with advanced or recurrent NSCLC receiving antibodies to PD-1 or to PD-L1 were enrolled for a retrospective study conducted at Kindai University Hospital, Kyoto University Hospital, and Izumi City General Hospital. Also, for the cohorts D and E, patients with advanced or recurrent NSCLC receiving cytotoxic chemotherapy without ICB therapy or TKIs as an initial therapy, respectively, were retrospectively enrolled at Kindai University Hospital and Kyoto University Hospital.

### Data collection.

For the Nivolution trial, clinical data were prospectively extracted and evaluated per the protocol. The data cutoff date was July 2017. For the validation cohort, medical records were reviewed retrospectively, and data regarding clinicopathologic features and treatment history were extracted. PFS was measured from treatment initiation to clinical or radiographic progression or death from any cause. Patients without documented clinical or radiographic disease progression were censored on the date of last followup. Tumor burden was clinically estimated by calculating baseline tumor size, which was quantified as the sum of the longest dimensions — minor axis for lymph node lesions and major axis for non–lymph node lesions — of all measurable target lesions, as previously described (44). The target lesions were reviewed on computed tomography or magnetic resonance imaging scans taken within 42 days before the start of first-line therapy.

### Analysis of soluble immune-checkpoint molecules.

Plasma levels of sPD-1, sPD-L1, and sCTLA-4 were measured with a fully automated immunoassay system (HISCL, Sysmex Corp.). The assay was conducted as previously described (36), with optimization to accommodate new antibodies. Antibodies used included: capture (clone M150-5, made in-house) and detection antibody (P-Rb-8, made in-house) for sPD-1; capture (clone 27A2, made in-house) and detection antibody (130021, Novus Biologicals) for sPD-L1; and capture (clone C-Rb-15, made in-house) and detection antibody (BNI3, Tonbo Biosciences) for sCTLA-4.

### IHC.

Protocol for IHC was conducted as previously described (3, 50). Details are described in Supplemental Methods.

### Gene expression analysis by RNA-Seq.

Whole-transcriptome analysis of tumor cells and whole-blood cells in the Nivolution trial was performed with an AmpliSeq Transcriptome Human Gene Expression Kit (Thermo Fisher Scientific). Protocols for RNA-Seq are described in Supplemental Methods.

### Flow cytometry.

Flow cytometry analysis was conducted as previously described (27). Details are described in Supplemental Methods.

### Microarray analysis of peripheral CD8^+^ T cells and gene enrichment analysis.

Protocols for microarray analysis of peripheral CD8^+^ T cells are described in Supplemental Methods. Differentially expressed genes were identified by the linear models for microarray analysis (limma) package of Bioconductor software (51). Gene enrichment analysis was performed with genes whose expression correlated with the plasma concentrations of soluble immune factors at *P* < 0.001. The analysis of the selected genes was conducted with the use of Metascape (http://metascape.org/gp/index.html#/main/step1) (52), with the “Express analysis” option. Feature genes representing naive, progenitor exhausted, or terminally exhausted CD8^+^ T cells were obtained from published data (46), and the correlation of their expression with the plasma concentrations of soluble immune factors was represented with a heat map. The heat map was constructed with the ggplot2 package of R studio (version 2022.2.2.485 with R version 4.2.0).

### Cytokine analysis.

Protocols for cytokine analysis are described in Supplemental Methods.

### Statistics.

Categorical and continuous variables were summarized descriptively as percentage and as median or mean ± SD values, respectively. Differences in continuous variables were assessed with the Mann-Whitney U test, and those in categorical variables with Fisher’s exact test. Correlations were examined with the Pearson correlation test, and the correlation coefficient |r| was evaluated. Differences in PFS curves constructed by the Kaplan-Meier method were assessed with the log-rank test, and the Cox proportional hazards regression model was adopted to determine HRs. CIs are at the 95% level, and statistical significance is defined as *P* < 0.05. *P* values for comparisons between 2 groups are 2 sided. Statistical analysis was performed with SPSS Statistics version 25 (IBM) or GraphPad Prism 7.0 (GraphPad Software).

### Study approval.

For cohort A (Nivolution trial), the trial protocol was approved by the IRB at each site, and the trial was performed in accordance with the provisions of the Declaration of Helsinki and with International Conference on Harmonization Good Clinical Practice Guidelines (trial registration number: UMIN000019674). All patients provided written informed consent before study entry. For the cohorts B to E, patients at Kyoto University Hospital, Kindai University Hospital, or Izumi City General Hospital were enrolled for a retrospective study that was conducted according to the Declaration of Helsinki and protocols approved by the IRB of each participating hospital. Patients provided written informed consent where applicable, or such informed consent was waived by IRB-approved protocols for aggregate deidentified data analysis.

### Data availability.

All of the data and methods are presented in the manuscript or in the Supplemental Materials. All individual values for figures and tables are shown in the Supplemental Supporting Data Values file. Microarray data have been deposited under GEO accession number GSE242860.

## Author contributions

T Honjo, KC, and HH conceived the study. K Nakagawa, T Honjo, KC, and HH secured funding. HH, KC, ST, RH, and T Hirano performed data analysis. HH and TK reviewed the clinical data and performed data analysis. HH, KF, TO, K Haratanai, TT, JT, TY, TI, KT, MT, HY, HO, YS, and T Hirai accrued patients. HH, TK, and K Haratanai supervised biospecimen sample collection. HH and YC performed statistical analysis. KC, RH, and T Hirano conducted microarray analysis of blood samples. KS and K Nishio supervised RNA-Seq analysis of tumor and blood samples. MG, K Higuchi, HU, and CS conducted and supervised soluble immune factor analysis by HISCL. HH, KC, TK, YT, and JT wrote the study protocol. HH and KC wrote the manuscript. All authors discussed the results.

## Supplementary Material

Supplemental data

ICMJE disclosure forms

Supplemental tables 3-4

Supporting data values

## Figures and Tables

**Table 1:**
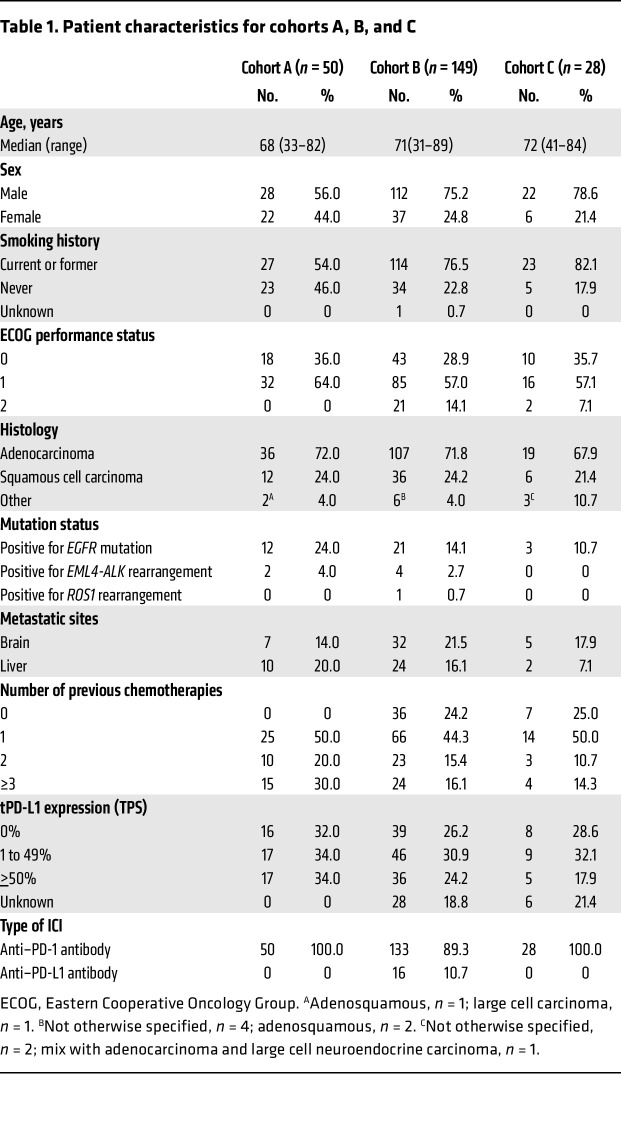
Patient characteristics for cohorts A, B, and C

## Version 1 04/01/2024

Electronic publication
