# Supplementary material for: Soluble immune checkpoint factors reflect exhaustion of antitumor immunity and response to PD-1 blockade
Source: J Clin Invest. 2024 Apr 1;134(7):e168318. doi: 10.1172/JCI168318 (PMC10977985; doi:10.1172/JCI168318)
Supplement: Supplemental data [file jci-134-168318-s216.pdf]

List of Supplemental Material

Supplemental Methods

Supplemental Figures 1 to 15

Supplemental Tables 1 to 4

Supplemental Methods

Study design and patients

The overall design of the study is outlined in Figure 1. From December 2015 to September 2016, 50 previously treated patients with advanced or recurrent NSCLC were prospectively enrolled in a phase 2 biomarker-finding trial, Nivolution, that was conducted at Kindai University Hospital. Patients were eligible for enrollment if an archival tumor tissue specimen obtained within 1 year before enrollment or newly biopsied tissue was available. Nivolumab (3 mg/kg) was administered intravenously biweekly. Radiologic imaging was performed every 6 weeks. Tumor response was assessed according to Response Evaluation Criteria in Solid Tumors (RECIST) version 1.1 (1). The study protocol was approved by the ethics committee at Kindai University Hospital and Kyoto University Hospital. Each patient provided written informed consent before enrollment.

For the cohorts B and C, patients with advanced or recurrent NSCLC receiving antibodies to PD-1 or to PD-L1—including nivolumab, pembrolizumab, and atezolizumab—were enrolled for a retrospective study conducted at Kindai University Hospital, Kyoto University Hospital and Izumi City General Hospital. Also, for the cohort D and E, patients with advanced or recurrent NSCLC receiving cytotoxic chemotherapy without ICB therapy or TKIs as an initial therapy, respectively, were retrospectively enrolled at Kindai University Hospital and Kyoto University Hospital. Blood samples and medical records were obtained for all patients. Tumor response was assessed by computed tomography every 6 to 12 weeks according to RECIST version 1.1. These studies were conducted according to the Declaration of Helsinki, and the protocols were approved by the Institutional Review Board of each hospital.

Immunohistochamistry

Tumor histology was classified according to WHO criteria (2). In the Nivolution trial, sections of formalin-fixed paraffin-embedded tumor tissue were subjected to IHC with monoclonal antibodies to PD-L1 (kit with clone 28-8, Abcam) and to CD8 (clone C8/144B, Dako). The percentage of tumor cells positive for PD-L1 (tPD-L1) was determined as previously described (3, 4). TILs were evaluated on the basis of staining for CD8. Tumor tissue samples including at least 100 viable tumor cells were eligible for assessment of TILs. The number of TILs was determined at an absolute magnification of 400× (0.20 mm^2 per field). At least one and a maximum of five scanned fields of tumor regions were randomly chosen for each TIL count. TILs were counted by a board-certified pathologist, and the density of TILs in each tumor was

calculated by dividing the number of TILs by the viewed fields (4). The cutoff value of 12.0/field was determined on the basis of the median number of tumor-infiltrated CD8$^+$ T cells per field.

Gene expression analysis by RNA-seq

The RNA extracted from tissue samples and blood cells was subjected to reverse transcription with the use of a SuperScript VILO cDNA Synthesis Kit (Thermo Fisher Scientific), and the resulting cDNA was subjected to multiplex PCR amplification, end repair, and ligation of barcoded adaptors. Pooled libraries were processed with an Ion Chef System (Thermo Fisher Scientific) for template preparation. Libraries were then loaded onto an Ion 550 chip and sequenced with the Ion S5 XL sequencing system. Ion Torrent Suite v5.10 software (Thermo Fisher Scientific) was used for base calling, alignment to the human reference genome (hg19), and quality control. Raw reads were analyzed automatically with the AmpliSeqRNA plugin to generate gene-level expression values for all 20,802 RefSeq human genes.

Flow cytometry

Fresh PBMCs were isolated from blood by Ficoll (EG Healthcare) density gradient centrifugation and were immediately stained with antibodies to CD8a (RPA-T8, Tonbo), to CD8 (SK1, Tonbo), and to PD-1 (EH12.2H7, BioLegend). Discrimination between live and dead cells was performed by staining with 7-aminoactinomycin D (7-AAD) (Tonbo, 13-6993), and data were gated on live (7AAD-negative) and single cells. Acquisition of samples was performed with a BD FACSCanto II cell analyzer (BD Biosciences). Data were collected with the use of BD FACSDiva software version 6.1.3 and further analyzed with FlowJo 10.4 (Tree Star).

Microarray analysis of peripheral CD8$^+$ T cells and gene enrichment analysis

CD8$^+$ T cells were purified from PBMCs with an AutoMACS system (Miltenyi Biotec). Total RNA was isolated from the cells with an RNeasy Micro Prep Kit (Qiagen), and its quality was analyzed with TapeStation (Agilent). Portions (5 ng) of the total RNA were labeled with the use of a GeneChip WT Pico Reagent Kit (Thermo Fisher Scientific) and subjected to hybridization with a Human GeneChip Clariom D Array (Thermo Fisher Scientific). The array data were analyzed with Signal Space Transformation–Robust Multichip Analysis (SST-RMA) and Sketch-Quantile normalization (Expression Console Software).

Cytokine analysis

Plasma samples were obtained by centrifugation of EDTA-treated whole blood at 2400 × *g* for 10 min at 4°C. Concentrations of the cytokines shown in Figure 6D were measured with V-PLEX Plus Proinflammatory Panel 1, Cytokine Panel 1, V-PLEX Plus Cytokine Panel 1 (Human), V-PLEX Plus Chemokine Panel 1 (Human), and Human ELISA Kits (Meso Scale Discovery Electrochemiluminescence Service). All assays were performed in triplicate. A correlation matrix for the plasma concentrations of the cytokines as well as those of sPD-1, sPD-L1, and sCTLA-4 was generated by Ward's clustering with squared Euclidean distances.

Determination of the cutoff values defining high versus low concentrations of each soluble factor

The cutoff values for soluble factor concentrations were determined with a proportional hazards model. A Cox proportional hazards model was thus fitted to the PFS data in order to estimate the HR for each covariate of interest. After sorting according to the biomarker values, dummy variables such as those shown in Supplemental Methods Table 1 below were generated.

Supplemental Methods Table 1

Time	Censor	Biomarker	DB(1)	DB(2)	DB(3)	DB(4)	DB(5)	DB(6)	DB(…)
12	1	21	0	0	0	0	0	0	..
1	1	23	1	0	0	0	0	0	…
7	1	24	1	1	0	0	0	0	…
1	0	25	1	1	1	0	0	0	…
10	1	26	1	1	1	1	0	0	…
7	1	27	1	1	1	1	1	0	…
10	1	28	1	1	1	1	1	1	…
…	…	…	…	…	…	…	…	…	…

The HR was then calculated from the proportional hazards model, with the explanatory variable being DB(X). The results are summarized in Supplemental Methods Table 2 below.

Supplemental Methods Table 2

Explanatory variable	HR	log[HR]
DB(1)	…	…
DB(2)	…	…
DB(3)	…	…
DB(4)	…	…

DB(5)	…	…
DB(6)	…	…
DB(…)	…	…

From the latter table, DB(X) for which the absolute value of log[HR] is maximum was identified. The point in Supplemental Methods Table 1 corresponding to the identified DB(X) is the cutoff point. For example, if DB(4) gives the maximum absolute value of log[HR], the cutoff point is the value between 25 and 26 in Supplemental Methods Table 1.

Supplemental Methods Reference

1. Eisenhauer EA, Therasse P, Bogaerts J, Schwartz LH, Sargent D, Ford R, et al. New response evaluation criteria in solid tumours: revised RECIST guideline (version 1.1). *Eur J Cancer.* 2009;45(2):228-47.
2. Travis WD, Brambilla E, Nicholson AG, Yatabe Y, Austin JHM, Beasley MB, et al. The 2015 World Health Organization Classification of Lung Tumors: Impact of Genetic, Clinical and Radiologic Advances Since the 2004 Classification. *J Thorac Oncol.* 2015;10(9):1243-60.
3. Borghaei H, Paz-Ares L, Horn L, Spigel DR, Steins M, Ready NE, et al. Nivolumab versus Docetaxel in Advanced Nonsquamous Non-Small-Cell Lung Cancer. *N Engl J Med.* 2015;373(17):1627-39.
4. Haratani K, Hayashi H, Tanaka T, Kaneda H, Togashi Y, Sakai K, et al. Tumor immune microenvironment and nivolumab efficacy in EGFR mutation-positive non-small-cell lung cancer based on T790M status after disease progression during EGFR-TKI treatment. *Ann Oncol.* 2017;28(7):1532-9.

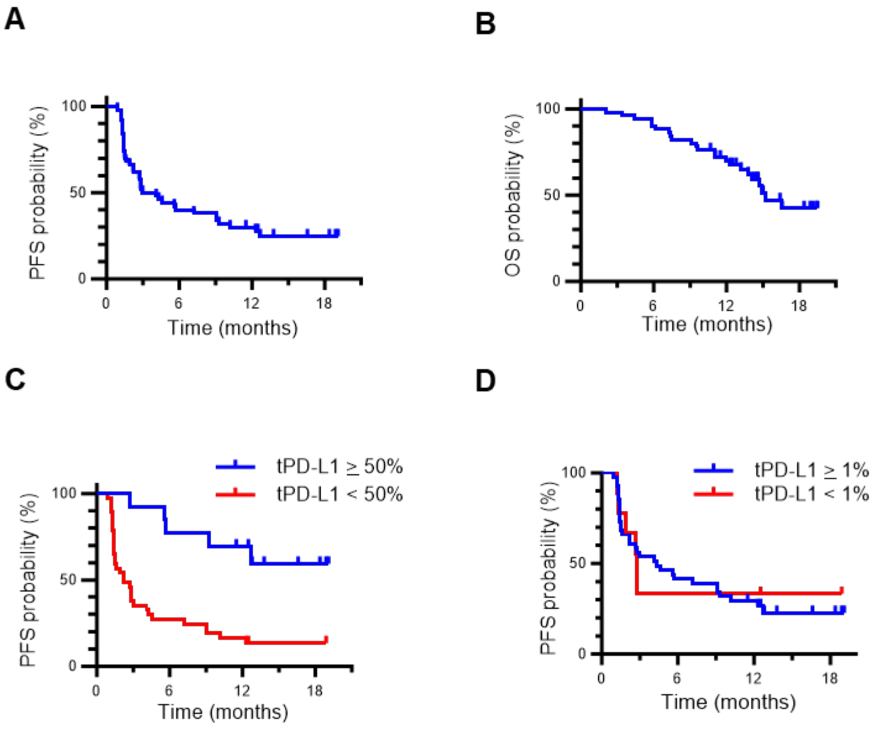

Supplemental Figure 1. Survival curves for patients in the Nivolution trial (cohort A). (**A** and **B**) Kaplan-Meier curves for PFS and overall survival (OS), respectively, for all 50 patients in the trial. (**C** and **D**) Kaplan-Meier curves for PFS according to high (*n* = 13 and 37, respectively) or low (*n* = 41 and 9, respectively) tPD-L1 based on cutoffs of 50% (**C**) or 1% (**D**). For the tPD-L1 cutoff of 50% (**C**) median PFS was not reached and 2.2 months for high and low tPD-L1, respectively (log-rank $P = 0.0004$), with an HR for high versus low tPD-L1 of 0.20 (95% CI, 0.08–0.53). For the tPD-L1 cutoff of 1% (**D**), median PFS was 4.3 and 2.8 months for high and low tPD-L1, respectively (log-rank $P = 0.88$), with an HR for high versus low tPD-L1 of 0.93 (95% CI, 0.40–2.20).

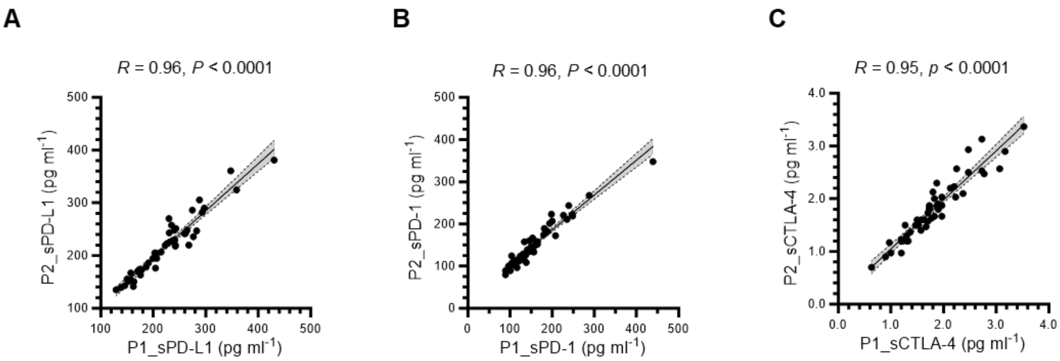

Supplemental Figure 2. Consistent detection of soluble immune factors in plasma of patients in the Nivolution trial before treatment. Pearson correlation between the concentrations of sPD-L1 (**A**), sPD-1 (**B**), or sCTLA-4 (**C**) measured in plasma obtained at time P1 (2 weeks to 72 h prior to the start of treatment) and at P2 (within 24 h prior to the start of treatment) for all 50 patients.

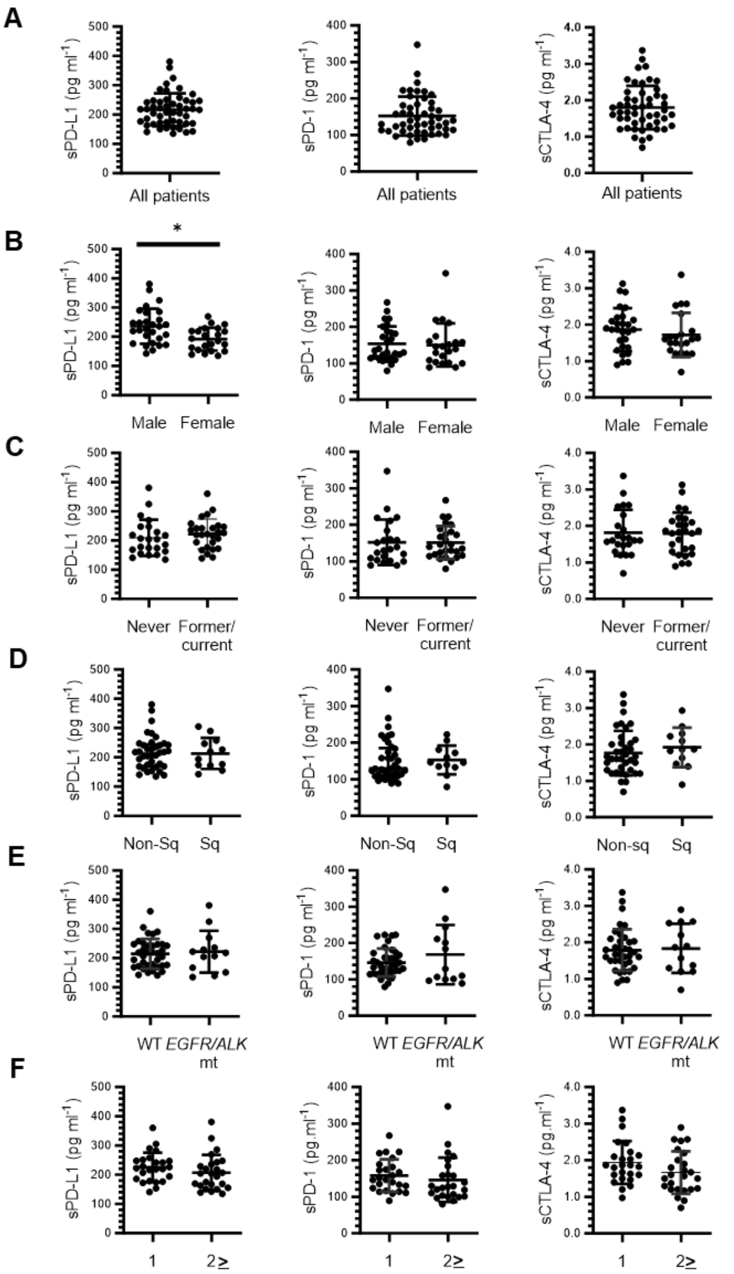

Supplemental Figure 3. Plasma concentrations of soluble immune factors according to patient characteristics for the Nivolution trial. (**A**) Concentrations of sPD-L1, sPD-1, and sCTLA-4 in plasma of all 50 patients. (**B**–**F**) Comparison of the levels of the soluble immune factors between patients classified according to sex (**B**), smoking status (**C**), histology (**D**), oncogenic driver mutations (**E**), or number of prior therapies (**F**). Sq, squamous cell carcinoma; non-Sq, non–squamous cell carcinoma; WT, wild type; *EGFR*, epidermal growth factor receptor gene; *ALK*, anaplastic

lymphoma kinase gene; mt, mutation. Mean ± SD values are indicated. *$P < 0.005$ (Mann-Whitney U test).

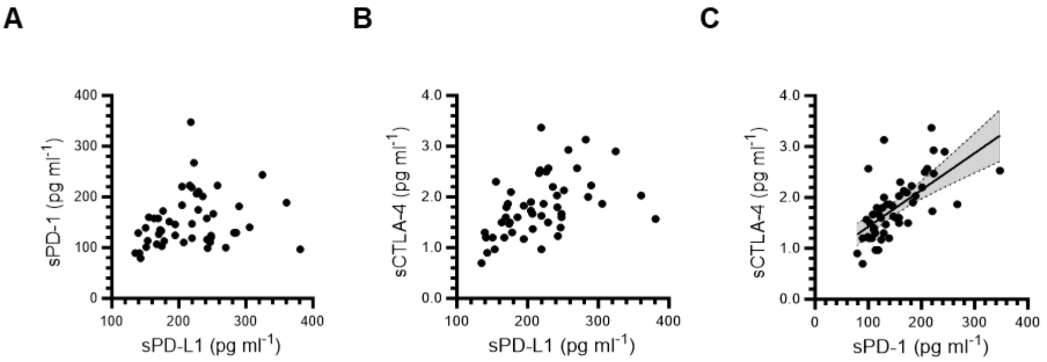

Supplemental Figure 4. Correlation analysis for plasma concentrations of each soluble immune factor for patients in the Nivolution trial. Pearson correlation for sPD-L1 versus sPD-1 (**A**), sPD-L1 versus sCTLA-4 (**B**), and sPD-1 versus sCTLA-4 (**C**) was determined for all patients (n = 50). For (**C**), the correlation was characterized by an R value of 0.64 and $P < 0.0001$; the gray shaded area above and below the solid line and bounded by the dotted lines indicates the 95% CI.

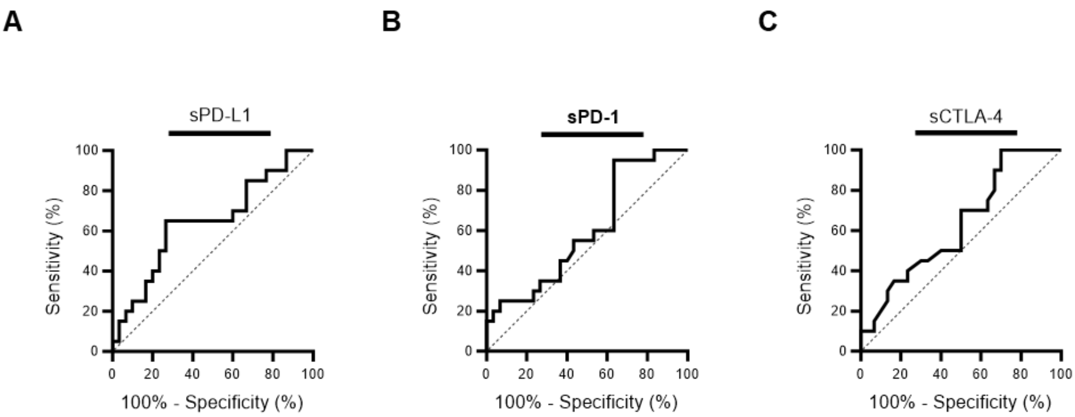

Supplemental Figure 5. ROC curve analysis for each soluble immune factor and prediction of 6-month PFS probability in the Nivolution trial. ROC curve analysis was performed for prediction of the 6-month PFS probability from the plasma concentrations of sPD-L1 (**A**), sPD-1 (**B**), or sCTLA-4 (**C**) for all patients.

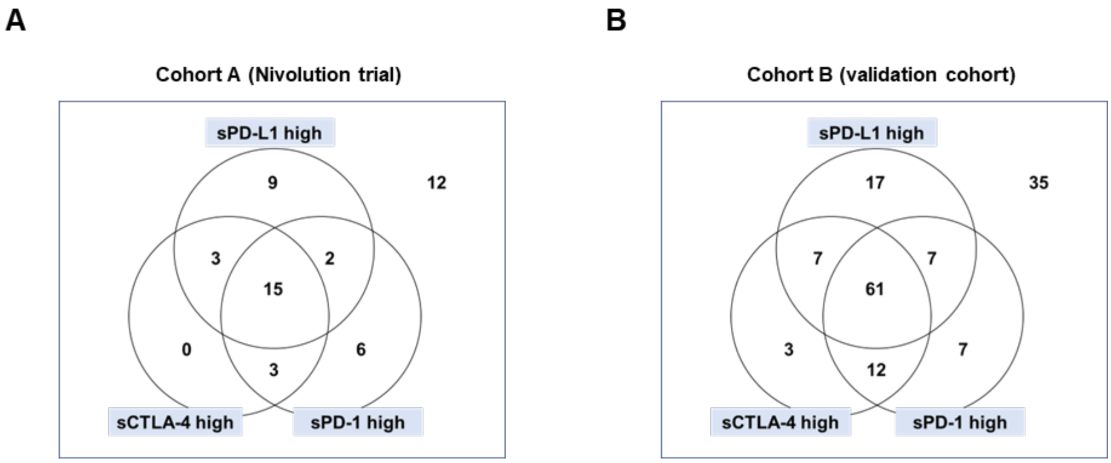

Supplemental Figure 6. Venn diagrams for patients with high levels of soluble immune factors. (**A**) Cohort A, Nivolution trial. (**B**) Cohort B (validation cohort).

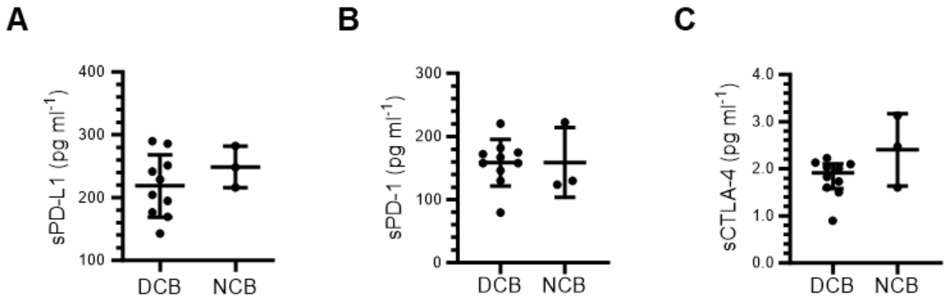

Supplemental Figure 7. Concentrations of soluble immune factors according to response for patients in the Nivolution trial with a tPD-L1 expression level of ≥50%. Plasma concentrations of sPD-L1 (**A**), sPD-1 (**B**), and sCTLA-4 (**C**) are compared between patients with a DCB (n = 10) or NCB (n = 3). Mean ± SD values are indicated. P values determined for the comparisons with the Mann-Whitney U test were not significant.

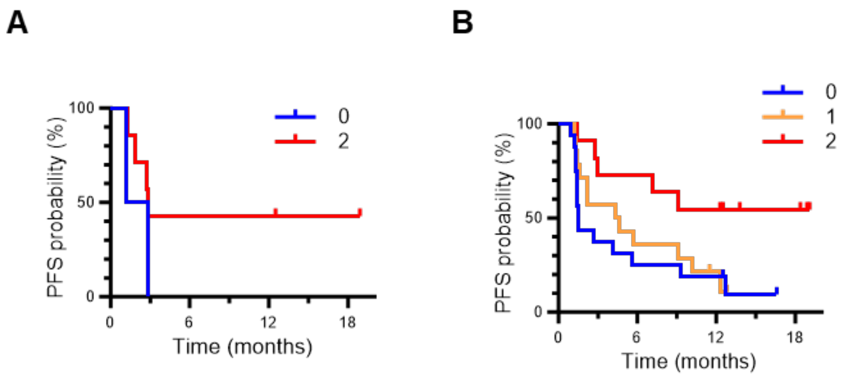

Supplemental Figure 8. Stratification of patients with a tPD-L1 expression level of <1% or ≥1% in the Nivolution trial according to the number of favorable immune factors. Kaplan-Meier curves for PFS are shown for patients with tPD-L1 expression levels of <1% (**A**) or ≥1% (**B**) according to the number of favorable immune factors defined as sCTLA-4 or sPD-L1 concentrations below the determined cutoff values (log-rank P = 0.29 and 0.03, respectively). Median PFS was 2.8 months, not evaluated, and 2.2 months for 2, 1, and 0 favorable factors, respectively, in (**A**), and not reached, 4.5 months, and 1.5 months, respectively, in (**B**). The HR for 1 (n = 0 and 14) versus 0 (n = 2 and 16) was not evaluated and 0.78 (95% CI, 0.36–1.70), and that for 2 (n = 7 and 11) versus 0 was 0.43 (95% CI, 0.05–3.50) and 0.30 (95% CI, 0.12–0.77), in (**A**) and (**B**), respectively.

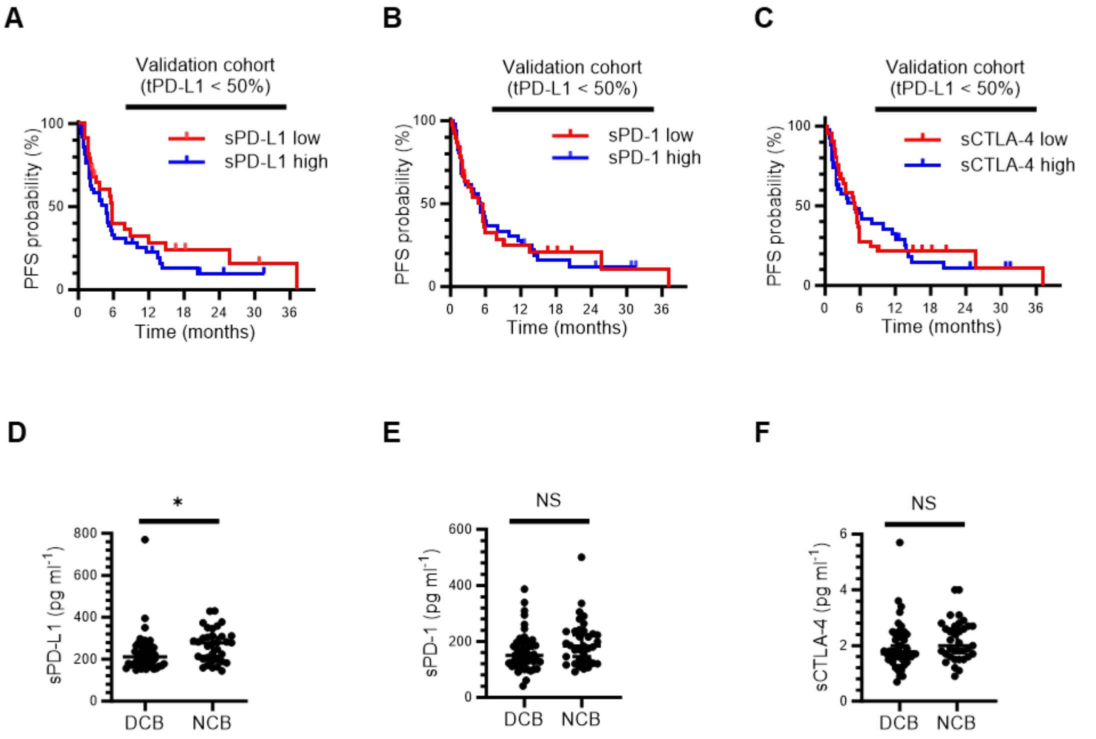

Supplemental Figure 9. (**A–C**) Kaplan-Meier curves for PFS of patients in the validation cohort (cohort B) with a tPD-L1 expression level of <50% according to high or low plasma concentrations of sPD-L1 (**A**), sPD-1 (**B**), or sCTLA-4 (**C**) based on the determined cutoff values. For sPD-L1 (high, n = 51; low, n = 34), median PFS was 5.8 versus 4.7 months for low and high sPD-L1, respectively (log-rank P = 0.18), with an HR of 0.76 (95% CI, 0.47–1.23). For sPD-1 (high, n = 46; low, n = 39), median PFS was 5.4 versus 5.1 months for low and high sPD-1, respectively (log-rank P = 0.98), with an HR of 1.12 (95% CI, 0.70–1.79). For sCTLA-4 (high, n = 42; low, n = 43), median PFS was 5.0 versus 5.1 months for low and high sCTLA-4, respectively (log-rank P = 0.82), with an HR of 1.07 (95% CI, 0.67–1.71). (**D–F**) Comparison of pretreatment plasma concentrations of sPD-L1 (**D**), sPD-1 (**E**), and sCTLA-4 (**F**) for patients in the validation cohort (cohort B) with a tPD-L1 level of <50% between those with a DCB (n = 50) or NCB (n = 35). Median ± 95% CI values are indicated. {*P < 0.05, NS (Mann-Whitney U test).

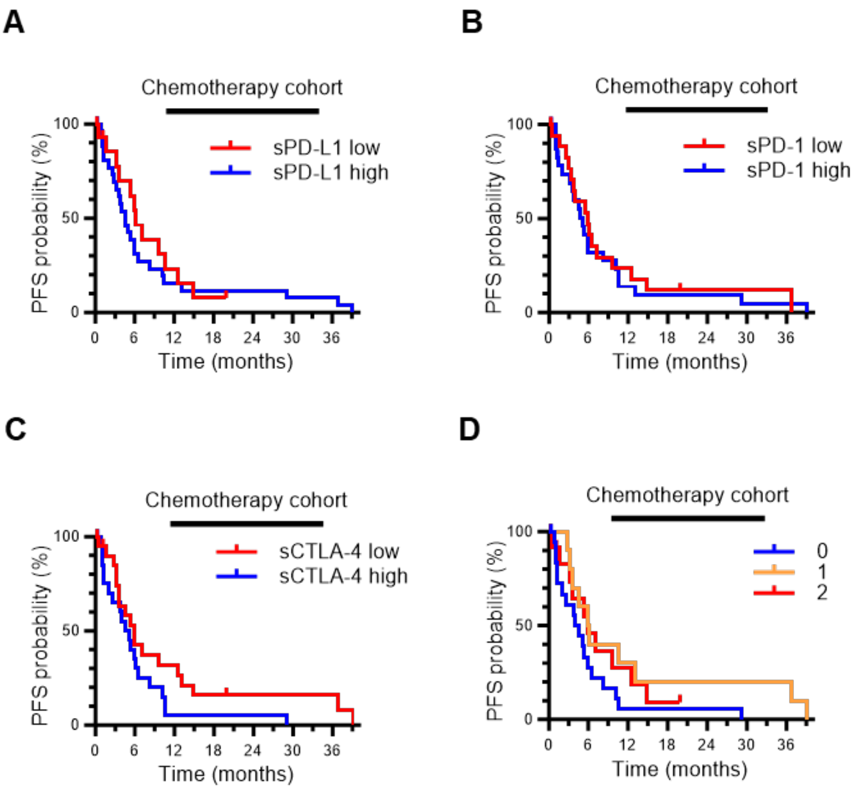

Supplemental Figure 10. PFS curves for patients treated with cytotoxic chemotherapy in a non-ICI cohort (cohort D). (**A**–**C**) Kaplan-Meier curves for PFS of patients treated with cytotoxic chemotherapy were determined according to high or low plasma concentrations of sPD-L1 (**A**), sPD-1 (**B**), or sCTLA-4 (**C**) based on the determined cutoff values. For sPD-L1 (high, $n = 27$; low, $n = 15$), median PFS was 6.1 versus 4.6 months for low and high sPD-L1, respectively (log-rank $P = 0.51$), with an HR of 0.75 (95% CI, 0.38–1.49). For sPD-1 (high, $n = 24$; low, $n = 18$), median PFS was 6.0 versus 5.1 months for low and high sPD-1, respectively (log-rank $P = 0.52$), with an HR of 0.86 (95% CI, 0.45–1.64). For sCTLA-4 (high, $n = 21$; low, $n = 21$), median PFS was 5.9 versus 4.9 months for low and high sCTLA-4, respectively (log-rank $P = 0.10$), with an HR of 0.57 (95% CI, 0.29–1.12). (**D**) Kaplan-Meier curves for PFS among patients according to the number of favorable immune factors defined as sCTLA-4 or sPD-L1 levels below the cutoff values (log-rank $P = 0.136$). Median PFS was 5.9, 6.0, and 4.3 months for 2, 1, and 0 favorable factors, respectively. The HR for 1 ($n = 10$) versus 0 ($n = 19$) was 0.45 (95% CI, 0.19–1.07), and that for 2 ($n = 13$) versus 0 was 0.61 (95% CI, 0.28–1.34).

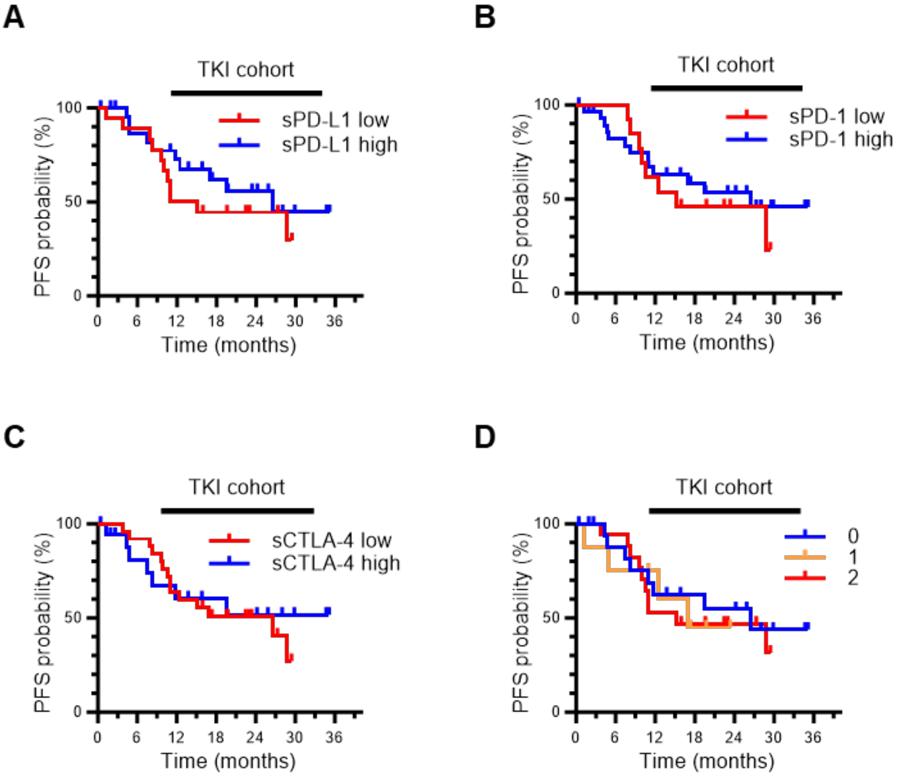

Supplemental Figure 11. PFS curves for patients treated with TKIs in a non-ICI cohort (cohort E). (**A**–**C**) Kaplan-Meier curves for PFS of patients treated with TKIs were determined according to high or low plasma concentrations of sPD-L1 (**A**), sPD-1 (**B**), or sCTLA-4 (**C**) based on the determined cutoff values. For sPD-L1 (high, $n = 25$; low, $n = 18$), median PFS was 13.1 versus 26.5 months for low and high sPD-L1, respectively (log-rank $P = 0.32$), with an HR of 1.49 (95% CI, 0.63–3.55). For sPD-1 (high, $n = 30$; low, $n = 13$), median PFS was 15.1 versus 26.5 months for low and high sPD-1, respectively (log-rank $P = 0.88$), with an HR of 1.29 (95% CI, 0.51–3.22). For sCTLA-4 (high, $n = 18$; low, $n = 25$), median PFS was 26.5 months versus not reached for low and high sCTLA-4, respectively (log-rank $P = 0.82$), with an HR of 1.16 (95% CI, 0.47–2.83). (**D**) Kaplan-Meier curves for PFS among patients according to the number of favorable immune factors defined as sCTLA-4 or sPD-L1 levels below the cutoff values (log-rank $P = 0.81$). Median PFS was 15.1, 16.9, and 26.5 months for 2, 1, and 0 favorable factors, respectively. The HR for 1 ($n = 8$) versus 0 ($n = 18$) was 1.49 (95% CI, 0.45–4.95), and that for 2 ($n = 17$) versus 0 was 1.43 (95% CI, 0.52–3.95).

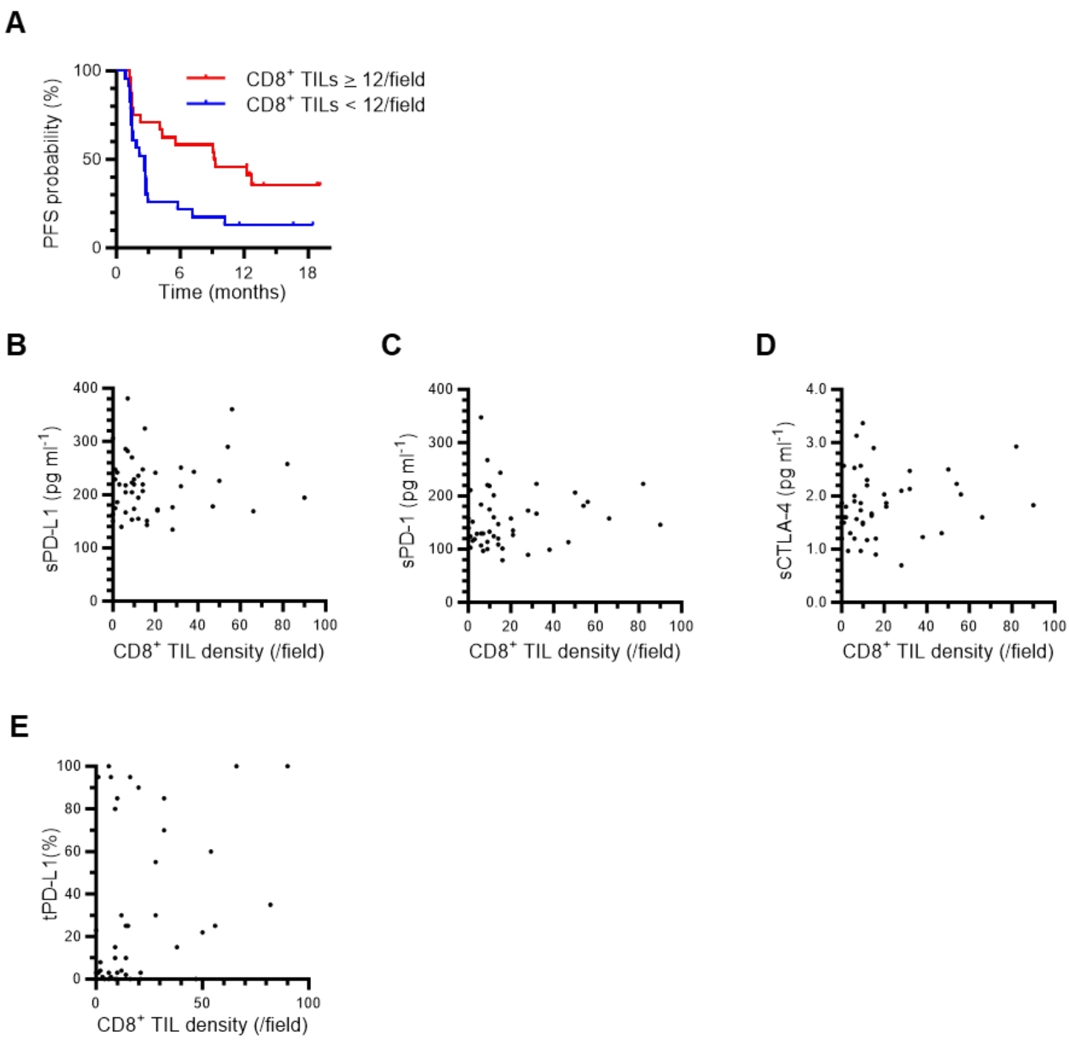

Supplemental Figure 12. Stratification of patients in the Nivolution trial according to CD8^{+} TIL density. (**A**) Kaplan-Meier curves for PFS of patients with hot or cold tumors defined on the basis of the number of CD8^{+} TILs [≥12.0/field (n = 23) or <12.0/field (n = 24), respectively]. Median PFS was 9.2 and 2.6 months for hot and cold tumors, respectively (log-rank P = 0.013), with an HR of 0.43 (95% CI, 0.22–0.86). (**B**–**E**) Pearson correlation of CD8^{+} TIL density and either plasma levels of sPD-L1 (**B**), sPD-1 (**C**), or sCTLA-4 (**D**) or tPD-L1 expression level (**E**) (n = 47).

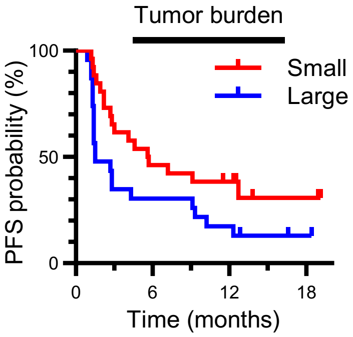

Supplemental Figure 13. Kaplan-Meier curves for PFS of patients in the Nivolution trial according to large or small tumor burden based on the median value. Median PFS was 5.7 versus 1.5 months for small (*n* = 26) and large (*n* = 23) tumor burden, respectively (log-rank *P* = 0.04), with an HR of 0.52 (95% CI, 0.27–1.02).

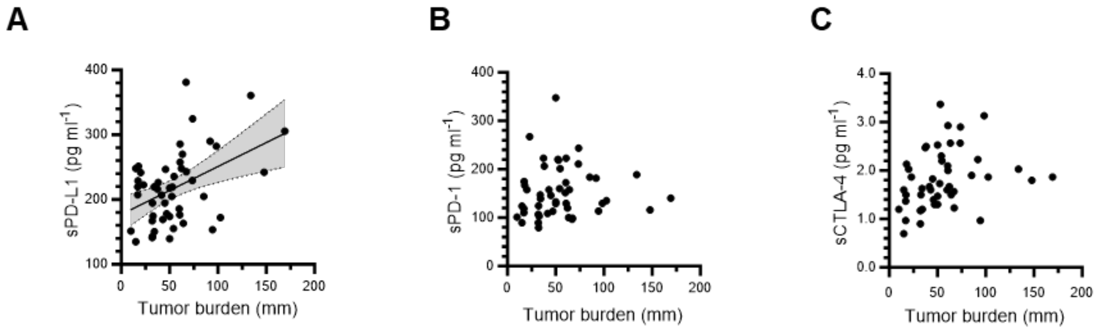

Supplemental Figure 14. Correlation between soluble immune factor concentrations and tumor burden for patients in the Nivolution trial. Pearson correlation was examined for plasma concentrations of sPD-L1 (**A**), sPD-1 (**B**), or sCTLA-4 (**C**) and tumor burden (n = 49). A moderate correlation is apparent in (**A**), with an R value of 0.46 and P = 0.0013; the gray shaded area above and below the solid line and bounded by the dotted lines indicates the 95% CI.

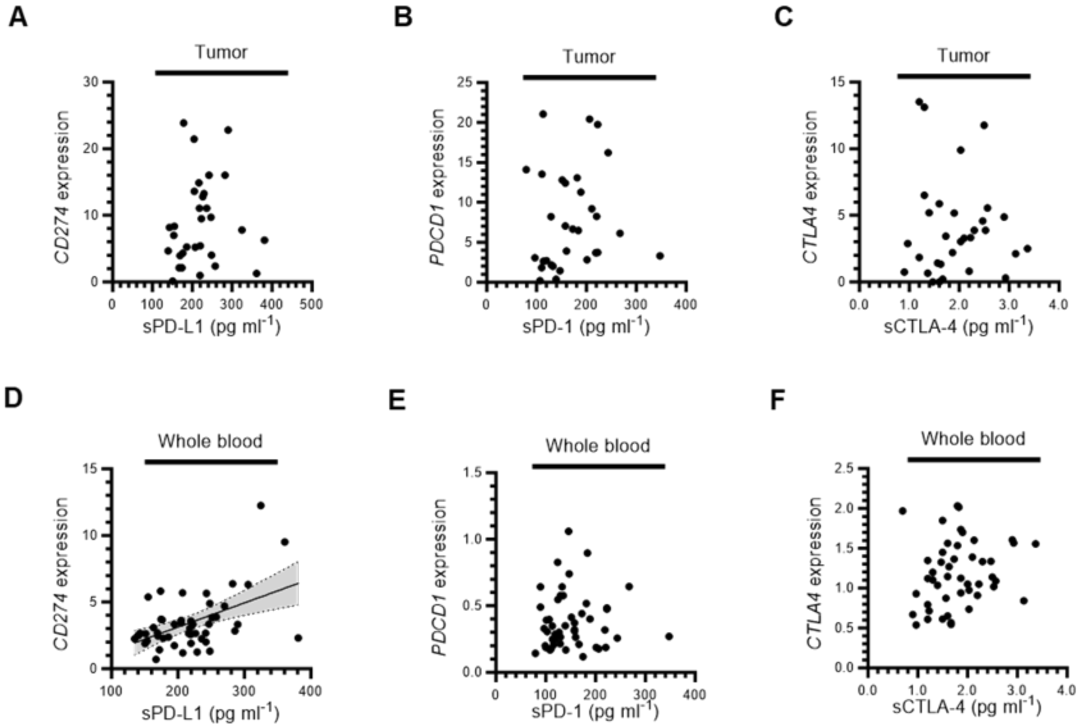

Supplemental Figure 15. Correlation between soluble immune factor concentrations and expression of the corresponding genes for patients in the Nivolution trial. Pearson correlation was examined for plasma levels of sPD-L1 (**A** and **D**), sPD-1 (**B** and **E**), or sCTLA-4 (**C** and **F**) and expression levels of the corresponding genes in tumor tissue (n = 31) (**A**–**C**) or whole-blood cells (n = 48) (**D**–**F**). A moderate correlation was apparent in (**D**), with an R value of 0.50 and $P < 0.001$; the gray shaded area above and below the solid line and bounded by the dotted lines indicates the 95% CI.

Supplemental Table 1. Patient characteristics for cohort D (cytotoxic chemotherapy cohort) and cohort E (TKI cohort)

	Cohort D (cytotoxic chemotherapy, n = 42)		Cohort E (TKI, n = 43)	
	No.	%	No.	%
Age, years				
Median (range)	69.5(33-85)		71 (40-83)	
Sex				
Male	32	76.2	24	55.8
Female	10	23.8	19	44.2
Smoking history				
Current or former	34	78.6	19	44.2
Never	9	21.4	24	55.8
Unknown	0	0	1	0.7
ECOG performance status				
0	24	57.1	14	32.6
1	17	40.5	23	53.5
2	1	2.4	3	7.0
3	0	0	2	4.7
Unknown	0	0	1	2.3
Histology				
Adenocarcinoma	32	76.2	42	97.7
Squamous cell carcinoma	10	23.8	1	2.3
Other	0	0	0	0
Mutation status				
None	34	81.0	0	0
Positive for *EGFR* mutation	4	9.5	43	100.0
Positive for *EML4-ALK* rearrangement	2	4.8	0	0
Other	2[A]	4.8	0	0
Type of treatment				
Platinum agent plus pemetrexed	25	59.5	36	0
Platinum agent plus taxane	14	33.3	66	0
Nonplatinum monotherapy	3	7.1	23	0
EGFR-TKIs	0	0	43	100.0

ECOG, Eastern Cooperative Oncology Group.

[A]*BRAF* mutation, n = 1; *MET* skipping mutation, n = 1.

Supplemental Table 2. Multivariate analysis of predictive factors for nivolumab efficacy in the Nivolution trial

Variable	Coefficient	95% CI	*P* value
Sex	0.96	0.45–2.03	0.91
Age	1.01	0.97–1.04	0.51
Histology	1.22	0.50–2.94	0.66
Driver mutation (*EGFR/ALK*)	1.26	0.46–3.44	0.66
tPD-L1 (TPS: <50% vs. ≥50%)	0.09	0.03–0.30	<0.001
Tumor burden	1.52	0.71–3.28	0.28
Number of favorable immune factors[A]	0.41	0.24–0.70	0.001

[A]Defined as sCTLA-4 or sPD-L1 concentrations below the determined cutoff values.

Conflict of interest:

HH has received support for the present study from Ono Pharmaceutical Co. Ltd. and Sysmex Corporation; honoraria from Amgen KK, AstraZeneca KK, Blueprint Medicines, Boehringer Ingelheim Japan Inc., Bristol-Myers Squibb Co. Ltd., Chugai Pharmaceutical Co. Ltd., Daiichi Sankyo Co. Ltd., Eli Lilly Japan KK, Janssen Pharmaceutical KK, Merck Biopharma Co. Ltd., MSD KK, Novartis Pharmaceuticals KK, Ono Pharmaceutical Co. Ltd., Sysmex Corporation, Taiho Pharmaceutical Co. Ltd., and Takeda Pharmaceutical Co. Ltd.; consulting fees from AstraZeneca KK, AbbVie GK, Boehringer Ingelheim Japan Inc., Bristol-Myers Squibb Co. Ltd., Chugai Pharmaceutical Co. Ltd., Daiichi Sankyo Co., Ltd. Janssen Pharmaceutical KK, Eli Lilly Japan KK, Guardant Health, Nippon Boehringer Ingelheim Co. Ltd., Novocure, Pfizer Japan Inc., Takeda Pharmaceutical Co. Ltd., and Merck Biopharma Co. Ltd.; and research funding from IQVIA Services JAPAN KK, Eisai Co. Ltd., SYNEOS HEALTH CLINICAL KK EP-CRSU CO., LTD., EPS Corporation., Shionogi & Co. Ltd., Nippon Kayaku Co. Ltd., Otsuka Pharmaceutical Co. Ltd., Takeda Pharmaceutical Co. Ltd., GlaxoSmithKline KK, MSD KK, Sanofi KK, Amgen Inc., Chugai Pharmaceutical Co. Ltd., Taiho Pharmaceutical Co. Ltd, Nippon Boehringer Ingelheim Co. Ltd., Bristol Myers Squibb Company, SRL Medisearch Inc., Janssen Pharmaceutical KK, PRA Health Sciences Inc., CMIC CO. Ltd., Astellas Pharma Inc. Pfizer R&D Japan GK, Ascent Development Services, Labcorp Development Japan KK, Eisai Inc., Kobayashi Pharmaceutical Co. Ltd., Bayer Yakuhin Ltd., and Pfizer Japan Inc.

KC has received research funding from Meiji Seika Pharma Co. Ltd., Meiji Holdings Co. Ltd., Shimazu Corporation, and Menarini Biomarkers Singapore; honoraria from Cosmo Bio Co. Ltd., Bristol Myers Squibb Japan, Merck KGaA, AstraZeneca KK, CHUGAI PHARMACEUTICAL CO. LTD., Novartis Pharma KK, Hitachi Ltd., Corning Incorporated, Agilent Technologies Japan Ltd., and SBI Pharmaceuticals Co. Ltd.

YT received honoraria from Ono Pharmaceutical Co. Ltd., AstraZeneca KK, Bristol-Myers Squibb Co. Ltd., Chugai Pharmaceutical Co. Ltd. MSD KK, Ono Pharmaceutical Co. Ltd, and Eisai KK; research funding from Daiichi-Sankyo, KORTUC, Janssen Pharma, and AstraZeneca; consultation fee from SONIRE Therapeutics Inc.

KF received honoraria from Chugai Pharmaceutical Co. Ltd. and KYORIN Pharmaceutical Co. Ltd.

ST received honoraria from Illumina, Inc. and NanoString Technologies.

K Haratani received honoraria from AS ONE Corporation and AstraZeneca KK and research funding from AstraZeneca KK.

TT received honoraria from AstraZeneca KK, Chugai Pharmaceutical Co. Ltd., MSD KK, Merck Biopharma Co. Ltd., Novartis Pharma KK, Takeda Pharmaceutical Co. Ltd., Taiho Pharmaceutical Co. Ltd., and Roche diagnostics.

JT received honoraria from AbbVie GK, AstraZeneca KK, Boehringer-Ingelheim Japan Inc., Bristol-Myers Squibb Co. Ltd., Chugai Pharmaceutical Co. Ltd, Daiichi sankyo Co. Ltd., Eli Lilly Japan KK, Janssen Pharmaceutical KK, MSD KK, Nihon Medi-Physics Co. Ltd, Nippon Kayaku Co. Ltd, Taiho Pharmaceutical Co. Ltd., Takeda Pharmaceuticals, Ono pharmaceutical Co. Ltd, and Pfizer Japan Inc.

TY received honoraria from Daiichi sankyo Co., Ltd., Pfizer Japan Inc., and Hisamitsu Pharmaceutical Co. Inc.

KT received honoraria from AstraZeneca KK, Merck Biopharma Co. Ltd., Eisai Co. Ltd., Bristol-Myers Squibb Co. Ltd., Ono pharmaceutical Co. Ltd, MSD KK, Chugai Pharmaceutical Co. Ltd, Takeda Pharmaceutical Co. Ltd., Taiho Pharmaceutical Co. Ltd., Merck Biopharma Co. Ltd., Novartis Pharma KK, and Kyowa Hakko Kirin Co. Ltd.

MT received honoraria from Chugai Pharmaceutical Co. Ltd, Novartis Pharma KK, AstraZeneca KK, Ono pharmaceutical Co. Ltd, Bristol-Myers Squibb Co. Ltd., Boehringer Ingelheim Japan Inc., and Takeda Pharmaceutical Co. Ltd.

HY received honoraria from Chugai Pharmaceutical Co. Ltd., MSD. KK, Bristol-Myers Squibb Co. Ltd., Ono Pharmaceutical Co. Ltd., and AstraZeneca KK; research funds from Chugai

Pharmaceutical Co. Ltd.

HO received honoraria from Chugai Pharmaceutical Co. Ltd., Sanofi KK, Eli Lilly Japan KK, MSD KK, AstraZeneca KK, Pfizer Japan Inc., Bristol Myers Squibb, and Takeda Pharmaceutical Co. Ltd.

KS received honoraria from Chugai Pharmaceutical Co. Ltd., Life Technologies Japan Ltd., Takeda Pharmaceutical Co. Ltd., Qiagen, Inc., Yodosha Co. Ltd, and Nippon Kayaku Co. Ltd.

MG, KH, HU, and CS are employees of Sysmex Corporation.

T Hirai received honoraria from Chugai Pharmaceutical Co. Ltd., Bristol-Myers Squibb Co. Ltd., and AstraZeneca KK.

K Nishio received honoraria from Boehringer Ingelheim Japan, Maruho Co. Ltd., AstraZeneca KK, Chugai Pharmaceutical Co. Ltd., Novartis Pharma KK, Eisai Co. Ltd., MSD KK, Bristol-Myers Squibb Co. Ltd., Ono pharmaceutical Co. Ltd, Pfizer Japan Inc., Sanofi SA, Guardant Health, Eli Lilly Japan KK, Amgen KK, Merck Biopharma Co. Ltd., Roche Diagnostics KK, Yakult Honsha Co. Ltd., Takeda Pharmaceutical Co. Ltd. Fujirebio Inc., Janssen Pharmaceutical KK; consulting fee from SymBio Pharmaceuticals KK, Eli Lilly Japan KK, and Otsuka Pharmaceutical Co. Ltd.; research funding from National Hospital Organization Osaka Minami Medical Center, Boehringer Ingelheim Japan Inc., West Japan Oncology Group, Thoracic Oncology Research Group, Nichirei Biosciences Inc. Eli Lilly Japan KK, Hitachi Life Technologies, and Sysmex Corporation.

K Nakagawa received honoraria from Ono Pharmaceutical Co. Ltd., Amgen Inc., Nippon Kayaku Co. Ltd., AstraZeneca KK, Chugai Pharmaceutical Co. Ltd., Eli Lilly Japan KK, MSD KK, Pfizer Japan Inc., Nippon Boehringer Ingelheim Co. Ltd., Taiho Pharmaceutical Co. Ltd., Bayer Yakuhin Ltd., CMIC ShiftZero KK, Life Technologies Japan Ltd., Neo Communication, Roche Diagnostics KK, AbbVie Inc., Merck Biopharma Co. Ltd., Kyowa Kirin Co. Ltd., Takeda Pharmaceutical Co. Ltd., 3H Clinical Trial Inc., Care Net Inc., Medical Review Co. Ltd., Medical Mobile Communications Co. Ltd, YODOSHA CO. LTD., Nikkei Business Publications Inc., Japan Clinical Research Operations, CMIC Co. Ltd., Novartis Pharma KK, TAIYO Pharma Co.

Ltd., KYORIN Pharmaceutical Co. Ltd., Bristol-Myers Squibb KK; consulting fees from Eli Lilly Japan KK, KYORIN Pharmaceutical Co. Ltd., Ono Pharmaceutical Co. Ltd., Pfizer Japan Inc.; patent planned for institution from Daiichi Sankyo Co., Ltd. (Patent no. US20200173999A1); research funding from AstraZeneca KK, MSD KK, Ono Pharmaceutical Co. Ltd., Nippon Boehringer Ingelheim Co. Ltd., Novartis Pharma KK, Pfizer Japan Inc., Bristol Myers Squibb Company, Eli Lilly Japan KK, Chugai Pharmaceutical Co. Ltd., Daiichi Sankyo Co. Ltd., Merck Biopharma Co. Ltd., PAREXEL International Corp., PRA HEALTHSCIENCES, EPS Corporation, Kissei Pharmaceutical Co. Ltd., EPS International Co. Ltd., Taiho Pharmaceutical Co. Ltd., PPD-SNBL KK, SymBio Pharmaceuticals Limited, IQVIA Services JAPAN KK, SYNEOS HEALTH CLINICAL KK, Nippon Kayaku Co. Ltd., EP-CRSU Co. Ltd., Mebix Inc., Janssen Pharmaceutical KK, AbbVie Inc., Bayer Yakuhin Ltd, Eisai Co. Ltd., Mochida Pharmaceutical Co. Ltd., Covance Japan Inc., Japan Clinical Research Operations, Takeda Pharmaceutical Co. Ltd., GlaxoSmithKline KK, Sanofi KK, Sysmex Corporation, Medical Reserch Support, Otsuka Pharmaceutical Co. Ltd.,SRL Inc., Pfizer R&D Japan GK, Amgen Inc.

T Honjo has received grant support for the present study from Ono Pharmaceutical Co. Ltd.; research grants from Sysmex Corp., Shimazu Corporation, Meiji Holdings Co. Ltd., Meiji Seika Pharma Co. Ltd., Menarini Biomarkers Singapore, and Bristol-Myers Squibb Co. Ltd.; and royalties from Ono Pharmaceutical Co. Ltd.

KC, HH, K Nakagawa, and T Honjo are authors on Patent: B 6719117, Method, reagent kit, device and computer program for assisting determination of efficacy of immune checkpoint inhibitor.
